# Testing plastomes and nuclear ribosomal DNA sequences as the next-generation DNA barcodes for species identification and phylogenetic analysis in *Acer*

**DOI:** 10.1186/s12870-024-05073-w

**Published:** 2024-05-23

**Authors:** Ning Fu, Yong Xu, Lu Jin, Tian-Wen Xiao, Feng Song, Hai-Fei Yan, You-Sheng Chen, Xue-Jun Ge

**Affiliations:** 1 Key Laboratory of Plant Resources Conservation and Sustainable Utilization, South China Botanical Garden, Chinese Academy of Sciences, Guangzhou, 510650 China; 2 University of Chinese Academy of Sciences, Beijing, 100049 China; 3 Conghua Middle School, Guangzhou, 510920 China

**Keywords:** *Acer*, Plastome, nrDNA, Next-generation DNA barcodes, Phylogeny

## Abstract

**Background:**

*Acer* is a taxonomically intractable and speciose genus that contains over 150 species. It is challenging to distinguish *Acer* species only by morphological method due to their abundant variations. Plastome and nuclear ribosomal DNA (nrDNA) sequences are recommended as powerful next-generation DNA barcodes for species discrimination. However, their efficacies were still poorly studied. The current study will evaluate the application of plastome and nrDNA in species identification and perform phylogenetic analyses for *Acer*.

**Result:**

Based on a collection of 83 individuals representing 55 species (c. 55% of Chinese species) from 13 sections, our barcoding analyses demonstrated that plastomes exhibited the highest (90.47%) species discriminatory power among all plastid DNA markers, such as the standard plastid barcodes *matK* + *rbcL* + *trnH*-*psbA* (61.90%) and *ycf1* (76.19%). And the nrDNA (80.95%) revealed higher species resolution than ITS (71.43%). *Acer* plastomes show abundant interspecific variations, however, species identification failure may be due to the incomplete lineage sorting (ILS) and chloroplast capture resulting from hybridization. We found that the usage of nrDNA contributed to identifying those species that were unidentified by plastomes, implying its capability to some extent to mitigate the impact of hybridization and ILS on species discrimination. However, combining plastome and nrDNA is not recommended given the cytonuclear conflict caused by potential hybridization. Our phylogenetic analysis covering 19 sections (95% sections of *Acer*) and 128 species (over 80% species of this genus) revealed pervasive inter- and intra-section cytonuclear discordances, hinting that hybridization has played an important role in the evolution of *Acer*.

**Conclusion:**

Plastomes and nrDNA can significantly improve the species resolution in *Acer*. Our phylogenetic analysis uncovered the scope and depth of cytonuclear conflict in *Acer*, providing important insights into its evolution.

**Supplementary Information:**

The online version contains supplementary material available at 10.1186/s12870-024-05073-w.

## Introduction

The accurate identification and description of species is a fundamental task in biology. Despite an estimated 10 million eukaryotic species globally, fewer than 3 million have been scientifically described [1, 2]. The discovery and description of these species require significant resources, including trained personnel and substantial investments of time and money. Even for species with scientific descriptions, traditional morphological methods for identifying unknown specimens can be challenging due to factors such as incomplete specimens, a shortage of taxonomists, or a lack of distinguishing features between species [3–5].

DNA barcoding, an approach to identifying species based on short DNA sequences, offers a solution to the challenges of traditional morphological classification. This approach has been widely studied and applied in animals due to its convenience and efficiency, with the mitochondrial sequence cytochrome oxidase I (*COI*) proving particularly useful as a DNA barcode [6–13]. However, the standard DNA barcodes used in plants, such as ITS, *rbcL*, *matK*, and *trnH*-*psbA*, do not consistently provide satisfactory species discrimination, especially for recently differentiated species [14–20].

The complete plastome and nuclear ribosomal DNA (nrDNA), which possess much more variable characters, have been recommended as next-generation barcodes (super barcodes/barcodes 2.0) [21–24]. Plastome and nrDNA, which also have multiple copies in each cell of plants, thus can be easily assembled from genome skimming data [15, 16, 25, 26]. With the ever-decreasing cost of genome skimming, more and more barcodes 2.0 have been generated from different plants [3, 27–33]. However, many of these studies only sampled one individual per species [28, 31, 32]. This approach is unable to reveal species boundaries because it fails to test species-level monophyly [3, 29]. Low species resolution from plastomes was sometimes reported, i.e., 27.27% in *Schima* [34], 28.6% in *Fargesia* [33], and c. 50% in *Rhododendron* [3], and chloroplast capture resulting from hybridization may be one of the main reasons for DNA barcoding failure in plants. The efficacy of barcodes 2.0 in more plant taxa, especially for those taxonomic challenging taxa, needs to be further assessed. Moreover, it is worth noting whether the addition of nrDNA can provide different insights from plastome, given the differences between their hereditary processes.

*Acer* L., also known as maple, is an economically important and species-rich genus with over 150 species globally [35, 36]. According to the widely accepted classification by de Jong [35], *Acer* species worldwide were divided into 19 sections. *Acer* is a taxonomic difficult genus, exhibiting abundant morphological variations due to the frequent interspecific/intraspecific hybridization/introgression [35, 37–47]. The morphological characteristics of inflorescence, leaf shape, bud scale, and fruit shape are highly variable among *Acer* species, and even among the conspecific individuals, there are significant differences in the morphology of vegetative organs [35, 37–40, 42, 44, 45]. An efficient DNA barcode is needed for precise species identification for *Acer* species.

Low species resolution was observed when utilizing several DNA barcodes, including *rbcL*, *matK*, *psbA*-*trnH*, *trnL*-*trnF*, *trnS-trnG*, ITS2, and ITS [37, 39, 48]. Lin et al. [37] reported a relatively high species resolution using ITS (73.09%); however, their sample size was limited to 52 individuals of 41 species, supplemented by 119 downloaded ITS sequences from only 10 species. Furthermore, they found ITS ineffective in discriminating species within sect. *Palmata* due to share identical sequences, indicating a shortage in interspecific variations. Similarly, Han et al. [39] reported a peak species resolution of 90.47% when combining four traditional barcodes (ITS + *rbcL* + *matK* + *trnS-trnG*); nevertheless, their study included only 18 *Acer* species (averaging 2 species per section), resulting in inadequate sampling representation within each section.

In recent years, several phylogenetic studies have acquired substantial progress by using plastomes or genome-wide data in *Acer* [49–52]. These studies both obtained highly supported phylogenies and revealed the phylogenetic relationships between *Acer* sections. Most notably, Li et al. [49] uncovered the phylogenetic relationships between 16 *Acer* sections based on 500 nuclear loci. Nevertheless, to our knowledge, no study has extensively compared the phylogenies generated from plastomes and large-scale nuclear sequences and visualized the comparison results for *Acer* so far. This hinders our further understanding of the evolution of this genus.

In this study, we applied a genome skimming approach to obtain whole plastomes and nrDNA of 83 individuals representing 55 *Acer* species. By evaluating the usefulness of plastome and nrDNA as barcodes 2.0 for this taxonomic difficult genus, we aim to address the following issue: (1) Compared to standard/taxon-specific DNA markers, can plastomes and nrDNA improve species discriminatory power in the genus *Acer*? (2) If so, to what extent and how do they enhance the discriminatory power? (3) What insights can plastomes provide into the evolution of *Acer*?

## Results

### Characteristics of *Acer* plastome

**Fig. 1:**
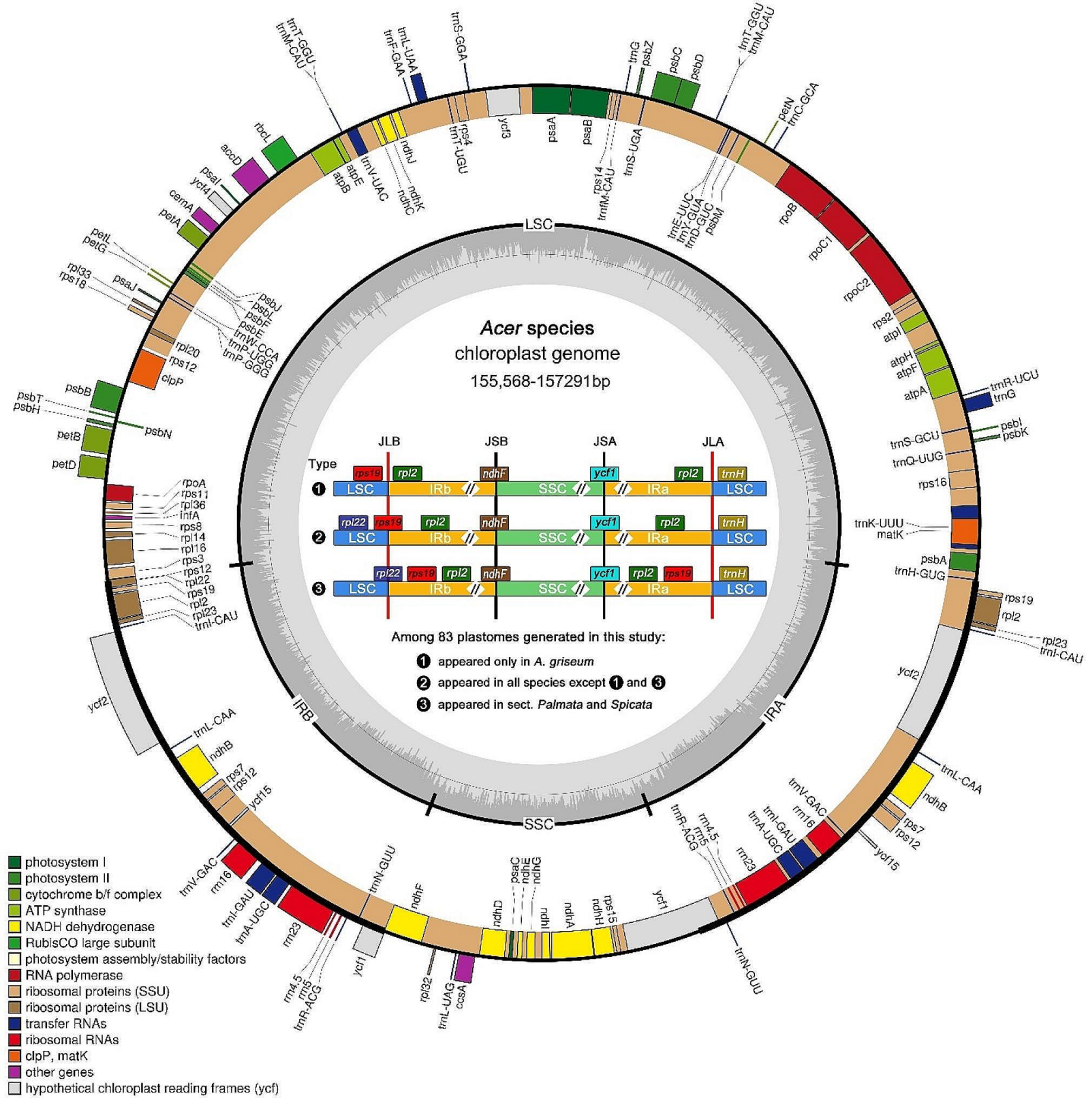
Plastome map of *Acer* species and three types of IR boundary identified in this study. Genes inside the outer circle are transcribed clockwise while those outside are transcribed counterclockwise. Genes are color-coded according to their function. Darker gray columns in the inner circle represent the GC content and the lighter gray columns accordingly correspond to the AT content

Complete plastomes of 83 accessions were successfully obtained without a gap. The size ranges from 155,568 bp (*A. carpinifolium* NJ216) to 157,291 bp (*A. confertifolium* GN100) (Table S1). All sequenced plastomes exhibited the typical quadripartite structure, consisting of a large single copy (LSC) region, a small single copy (SSC) region, and a pair of inverted-repeat (IR) regions (IRa and IRb) (Fig. 1). The overall GC content of these new sequences range from 37.9 to 38% (Table S1). Due to the presence of GC-rich rRNA, IR regions have the highest GC content (42.7–43%), which is higher than the LSC (36-36.2%) and the SSC (32.1–32.4%). All plastomes contain 82 protein-coding genes, 31 transfer RNA (tRNA) genes, and four ribosomal RNA (rRNA) genes (Table S2).

The comparative analysis of IR boundaries among 83 plastomes generated in this study uncovered three types of IR boundaries (Fig. 1). Type 1 only appears in *A*. *griseum*, while type 3 only exists in sect. *Palmata* and sect. *Spicata*; all the remaining *Acer* species exhibit type 2. From type 1 to type 3, a gradual expansion of the IRb region into the LSC region was observed. Previous studies reported that the expansion/contraction of IR borders could result in gene duplication/loss [53–55]. In this study, plastomes with a type 3 IR boundary harbor one more copy of gene *rps19* than the other two types due to the expansion of the IRb region into the LSC region, congruent with the results of previous studies [51, 56, 57]. In the study by Xia et al. [51], it was also found that the IR boundary of *A*. *griseum* is type 1. We also validated the boundary region of this species by aligning the NGS data against its plastome, confirming its existence (Figure S1). This type 1 boundary has also been reported in other species, such as *A*. *maximowiczii* in Areces-Berazain et al. [57], and *A*. *amplum* and *A*. *sterculiaceum* in Wang et al. [56]. However, in our study, these three species did not exhibit a type 1 IR boundary, and they have all been validated (Figure S1).

### Divergence hotspots

The five most variable regions were identified as divergent hotspots in the sliding window analysis (Figure S2). The most variable marker is *ndhC*-*trnV* (Pi = 0.02339), followed by *ndhF*-*trnL* (Pi = 0.02265), *trnK*-*rps16* (Pi = 0.01933), *trnS*-*trnfM* (Pi = 0.01889), *ycf1* (Pi = 0.01331) (Table S3). *Ycf1* had the highest percentage of variable sites (11.77%) and contained the most variable sites (513), as well as parsimony informative (PI) sites (291), while *ndhF*-*trnL* exhibited the highest percentage of PI sites (7.52%). The four most variable markers (*ndhC*-*trnV*, *ndhF*-*trnL*, *trnK*-*rps16*, and *trnS*-*trnfM*) were combined as a dataset to assess their discriminatory power for the following barcoding analysis. *Ycf1* showed relatively higher individual variation, with haplotypes up to 63, which is much higher than 55 (the number of sampled species in this study), thus it was separately evaluated for the barcoding analysis.

### Characteristics of different barcoding datasets

The plastome dataset (dataset A) was the largest among plastid datasets (dataset A-E), with an aligned length of 138,552 bp (Table 1). The nrDNA dataset (dataset F) had an aligned length of 6,773 bp, which is much longer than the ITS dataset (dataset G, 734 bp). Dataset H was the largest (145,325 bp) among all datasets as it combined the plastome dataset and nrDNA dataset.

**Table 1:** Feature comparison of different datasets

Data set	Data set code	Aligned length (bp)	Variable sites		PI sites		Haplotypes
			Number	%	Number	%	
Plastome	A	138,552	7,501	5.41	4,811	3.47	75
Coding region	B	76,307	2,903	3.80	1,815	2.38	70
Combined four most variable markers	C	6,511	629	9.66	433	6.65	63
*ycf1* (SSC portion)	D	4,359	513	11.77	291	6.68	63
*matK* + *rbcL + trnH-psbA*	E	3,615	225	6.22	148	4.09	48
nrDNA	F	6,773	368	5.43	297	4.39	68
ITS	G	734	159	21.66	131	17.85	58
Plastome + nrDNA	H	145,325	7,869	5.41	5,108	3.51	80

Note: PI: Parsimony informative sites

The plastome + nrDNA dataset (dataset H) had the largest number of variable sites (7,869) and PI sites (5,108) (Table 1). The plastome dataset (dataset A) contains 7,501 variable sites and 4,811 PI sites, much higher than that of the standard plastid barcodes (*matK + rbcL* + *trnH-psbA*, dataset E) (225 variable sites and 148 PI sites) and that of the taxon-specific hypervariable markers (dataset C and D). The nrDNA dataset (dataset F) had many more variable sites (368) and PI sites (297) than the ITS dataset (dataset G) (159 variable sites and 131 PI sites). Among all datasets, the ITS dataset (dataset G) (with 21.66% variable and 17.85% PI sites) exhibited the highest percentage of variable sites as well as PI sites, followed by *ycf1* (dataset D), then the combination of the four most variable markers (dataset C).

### Species discrimination

#### Species discrimination based on phylogenetic tree

**Fig. 2:**
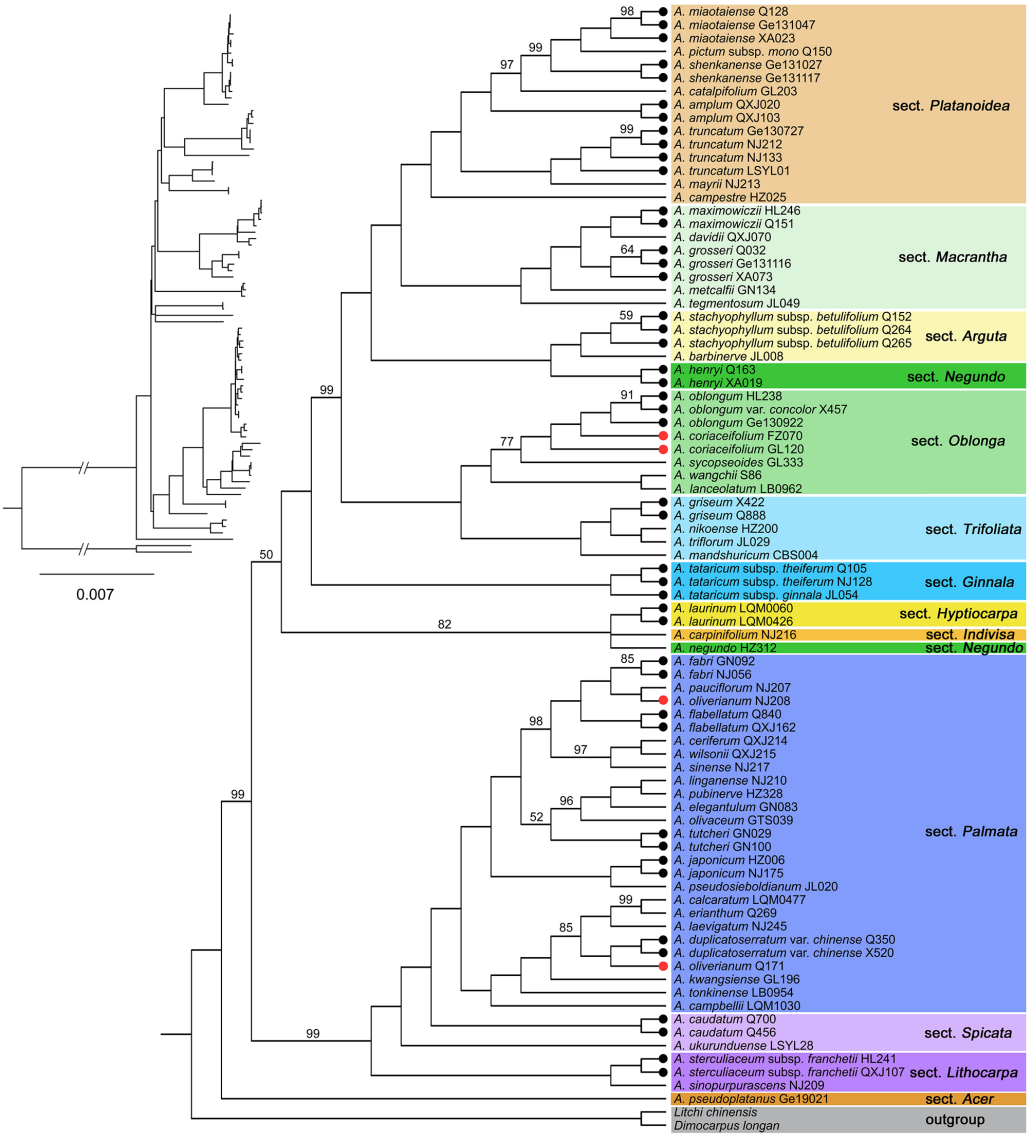
ML tree inferred from complete plastomes generated by this study. ML bootstrap support (BS) values are shown at nodes. Clades were set to polytomy when BS < 50%. Species with multiple individuals sampled were marked with dots at branch ends, with black indicating monophyly, while red indicating non-monophyly

**Fig. 3:**
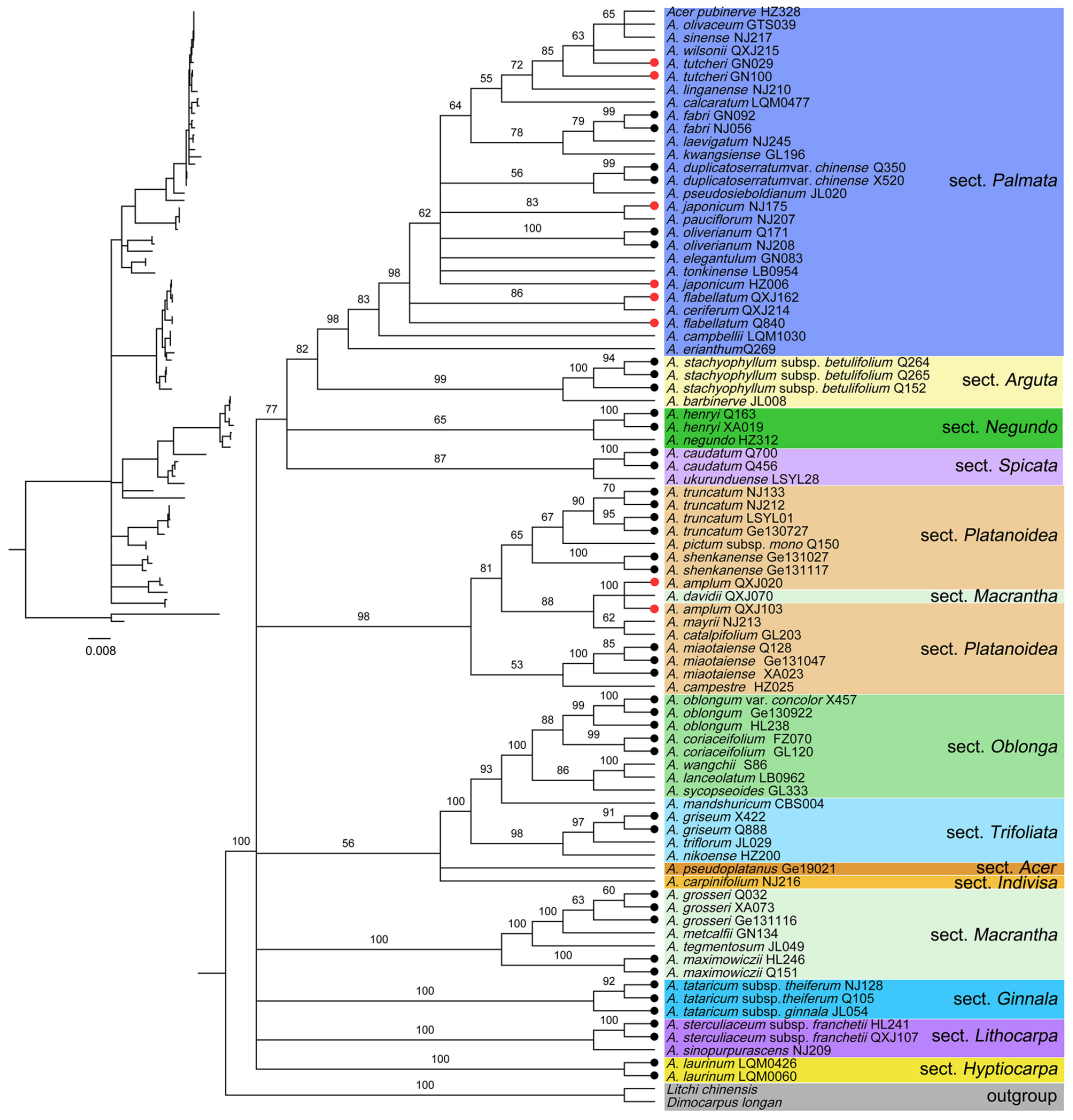
ML tree inferred from nrDNA generated by this study. ML bootstrap support (BS) values are shown at nodes. Clades were set to polytomy when BS < 50%. Species with multiple individuals sampled were marked with dots at branch ends, with black indicating monophyly, while red indicating non-monophyly

In the tree-based method, a species with all conspecific individuals resolved as monophyletic (with a support value ≥ 50%) was considered to be successfully identified. The plastome-wide datasets (datasets A and B) exhibited higher resolution than the standard plant barcodes (*matK + rbcL* + *trnH-psbA*, dataset E) and taxon-specific hypervariable markers (datasets C and D) for the 21 species with multiple individuals sampled (Table 2; Figs. 2 and 3, Figure S3). The plastome, coding region, and plastome + nrDNA (dataset H) datasets all showed the highest resolution of 90.47% (19/21 species successfully discriminated), followed by the combination of the four most variable markers (80.95%) and nrDNA (80.95%), *ycf1* (76.19%), ITS (66.67%), and *matK* + *rbcL* + *trnH*-*psbA* (61.90%).

**Table 2:** Comparison of species discriminatory efficiency between two methods

Data set	Data set code	Tree-based method	Distance-based method	0K2P	0K2P^55^	AMID
Plastome	A	90.47% (19/21)	90.47% (19/21)	0	0	220 (20 − 1,004)
Coding region	B	90.47% (19/21)	85.71% (18/21)	0	0	62 (5-292)
Combined four most variable markers	C	80.95% (17/21)	71.43% (15/21)	2	3	17 (0–71)
*ycf1* (SSC portion)	D	76.19% (16/21)	76.19% (16/21)	2	3	12 (0–59)
*matK* + *rbcL + trnH-psbA*	E	61.90% (13/21)	61.90% (13/21)	7	35	5 (0–28)
nrDNA	F	80.95% (17/21)	76.19% (16/21)	3	7	17 (0–71)
ITS	G	66.67% (14/21)	71.43% (15/21)	3	9	11 (0–45)
Plastome + nrDNA	H	90.47% (19/21)	90.47% (19/21)	0	0	212 (30–942)

Note: 0K2P: the total number of species (with multiple individuals) that failed to be discriminated due to showing minimum interspecific K2P distance of zero with other species; 0K2P^55^: the total number of species pairs with 0 K2P distance based on all 83 samples representing 55 *Acer* species; AMID: the average of minimum interspecific differences calculated from 21 *Acer* species with multiple individuals

#### Species discrimination based on K2P distance

In the distance-based method, a species with multiple individuals was regarded as successfully identified when it had a distinct barcoding gap, which means that its minimum interspecific distance is larger than its maximum intraspecific distance [58, 59]. The total number of barcoding gaps in eight datasets ranged from 13 to 19 (Figure S4, Table 2). On the whole, the distance-based method exhibited a similar tendency to the tree-based method. Among the eight datasets, both the plastome and plastome + nrDNA datasets had the highest resolution of 90.47%, followed by the coding region dataset (dataset B) (85.71%), both *ycf1* and nrDNA datasets were 76.19%, both the combined four most variable markers and ITS datasets were 71.43%, finally the *matK* + *rbcL* + *trnH-psbA* dataset was 61.90% (Table 2).

Among the 21 species with multiple individuals, no species failed to be discriminated because none showed a minimum interspecific K2P distance of zero in the plastome, coding region, and plastome + nrDNA datasets (Table 2). Furthermore, even among all 83 samples representing 55 species, there were also no species pairs showing 0K2P distance in these three datasets. In contrast, both datasets C and D had 3 pairs of species exhibiting 0K2P distance. For other datasets (datasets E-G), 7 to 35 pairs of species were found with 0K2P distance.

#### Comparison of species discriminatory power between plastome and standard plant barcodes

The plastome dataset significantly improved the species resolution compared to the standard plant barcodes. In the tree-based method, six species were additionally identified by the plastome dataset compared to the standard plant barcodes *matK* + *rbcL* + *trnH-psbA* (Table 3). These six species include four species of sect. *Palmata* (i.e., *A*. *fabri*, *A*. *flabellatum*, *A*. *japonicum*, *A*. *tutcheri*), *A*. *maximowiczii* of sect. *Macrantha*, and *A*. *oblongum* of sect. *Oblonga*.

**Table 3:** Comparison of species discriminatory power among four datasets in tree-based method

Species	Plastome		matK + rbcL + trnH-psbA		nrDNA		ITS
	Monophyly (BS%)		Monophyly (BS%)		Monophyly (BS%)		Monophyly (BS%)
*Acer amplum*	Y (100)		Y (78)		N		N
*A. caudatum*	Y (100)		Y (100)		Y (100)		Y (100)
*A. coriaceifolium*	N		N		Y (99)		Y (96)
*A. duplicatoserratum* var. *chinense*	Y (100)		Y (62)		Y (99)		Y (98)
*A. fabri*	Y (85)		N		Y (99)		N
*A. flabellatum*	Y (100)		N		N		N
*A. griseum*	Y (100)		Y (100)		Y (91)		N
*A. grosseri*	Y (100)		Y (95)		Y (63)		Y (69)
*A. henryi*	Y (100)		Y (100)		Y (100)		Y (100)
*A. japonicum*	Y (100)		N		N		N
*A. laurinum*	Y (100)		Y (100)		Y (100)		Y (100)
*A. maximowiczii*	Y (100)		N		Y (100)		Y (100)
*A. miaotaiense*	Y (100)		Y (62)		Y (100)		Y (100)
*A. oblongum*	Y (100)		N		Y (99)		Y (77)
*A. oliverianum*	N		N		Y (100)		Y (90)
*A. shenkanense*	Y (100)		Y (64)		Y (100)		Y (100)
*A. stachyophyllum* subsp. *betulifolium*	Y (100)		Y (100)		Y (100)		Y (100)
*A. sterculiaceum* subsp. *franchetii*	Y (100)		Y (94)		Y (100)		N
*A. tataricum*	Y (100)		Y (100)		Y (100)		Y (99)
*A. truncatum*	Y (100)		Y (85)		Y (90)		Y (86)
*A. tutcheri*	Y (100)		N		N		N

Note: BS%, bootstrap support value; N, no; Y, yes

The plastome also increased the support value when species were discriminated (Table 3). Among the 19 species that were successfully discriminated by the plastome dataset, 18 species obtained 100% support value, and *A*. *fabri* was supported at 85%. However, among the 13 species that were successfully identified by the *matK* + *rbcL* + *trnH-psbA* dataset, only six species were supported at 100%, while the support values of five species were below 90% (three species acquired support values below 65% when they were successfully identified).

### Phylogenetic analysis of *Acer*

**Fig. 4:**
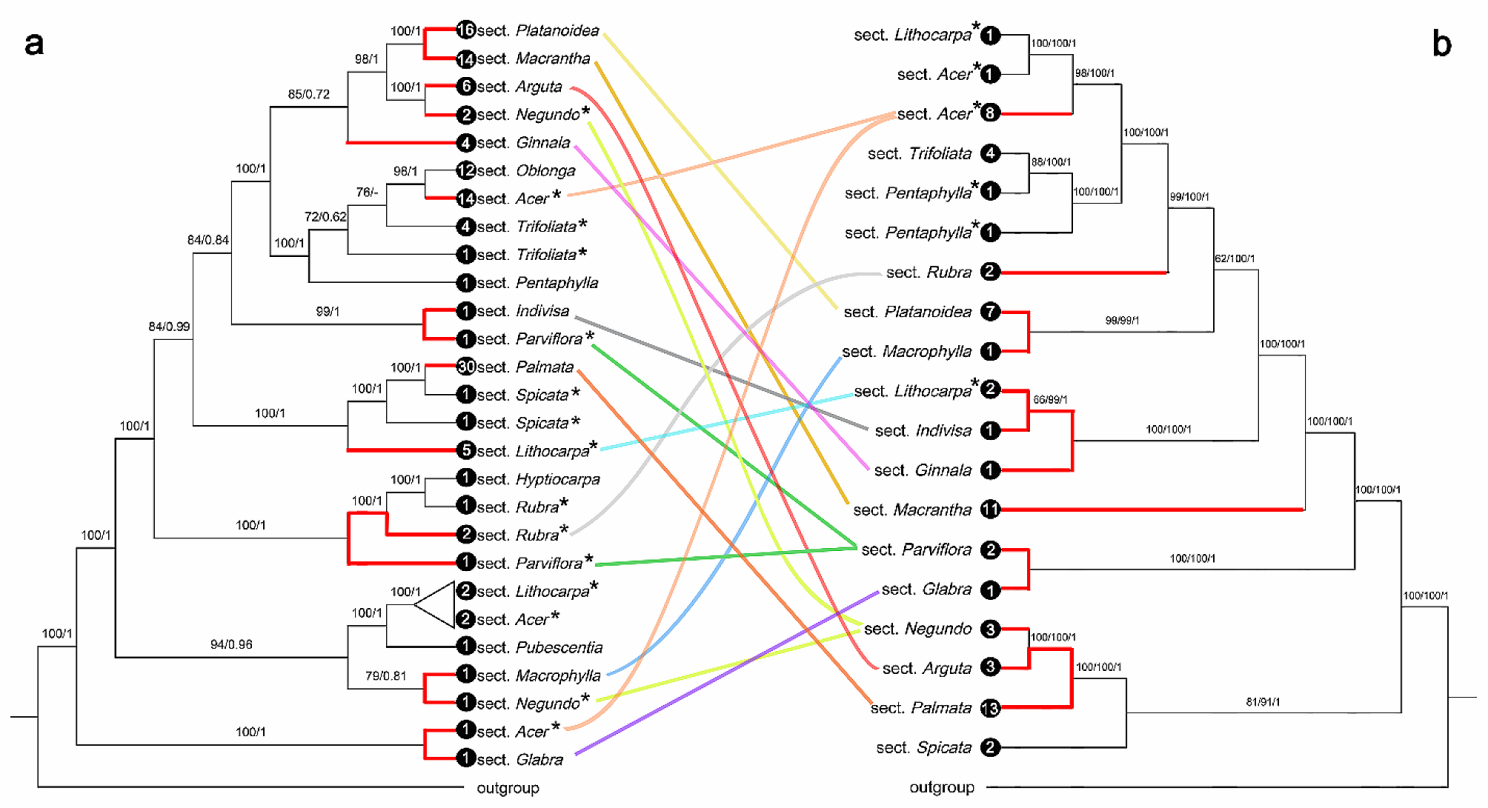
The comparison between (**a**) the plastid phylogeny generated by this study and (**b**) the phylogeny inferred from 500 nuclear loci by Li et al. (2019). The plastid phylogeny was integrated from the results of the partitioned and unpartitioned 80 CDSs datasets. Branches exhibiting obvious cytonuclear conflict were highlighted in red. Non-monophyletic sections were marked with an asterisk (*) behind their names. The number of sampled species of each branch was presented at the end of the branch. A branch where the species relationships conflict in the results of the two partitioning strategies was contracted

An ML tree containing 267 *Acer* plastomes (128 species and 19 sections) was first constructed (Figure S5). Based on this ML tree, we selected 128 representative accessions (one accession per species) for the following phylogenetic analysis. Using these 128 plastomes (128 species, c. 81% of *Acer* species), two datasets of 80 CDSs were constructed. For these two datasets, tree topologies generated from ML and BI analyses were consistent, and the partitioning strategy only had a slight effect on topology as well as the node support values of the phylogeny (Figure S6). We obtained a well-supported phylogenetic tree after integrating the results of these two datasets (i.e., retaining the higher supported clades) (Fig. 4a).

Comparing the resulting plastid phylogenetic tree with the phylogeny of Li et al. [49] based on 500 nuclear loci, we found many significant cytonuclear discordances between/within sections (see red branches in Fig. 4). Sect. *Platanoidea* and sect. *Macrantha* were 100% supported as sisters in our plastid phylogeny, however, they were quite distant in the nuclear phylogeny. Similar discordances also occurred in sects. *Indivisa* and *Parviflora*, sects. *Rubra* and *Parviflora*, sects. *Macrophylla* and *Negundo*, and sects. *Acer* and *Glabra*. In the nuclear phylogeny, sect. *Arguta* was closely related to sect. *Palmata*, but they were quite distantly related in the plastid phylogeny. And similar conflicts were also found between sects. *Parviflora* and *Glabra*, sects. *Indivisa*, *Lithocarpa* and *Ginnala*, sects. *Platanoidea* and *Macrophylla*. Moreover, we found that sects. *Negundo* and *Parviflora* were both monophyletic in the nuclear tree, however, they were both non-monophyletic with distantly related species in the plastid tree. In addition, although sect. *Acer* was non-monophyletic in both the plastid and nuclear trees, it also exhibited intra-section cytonuclear conflict.

## Discussion

### Comparison of species discriminatory power among different barcodes

Plastomes and nrDNA serving as barcodes 2.0 can effectively improve the species resolution compared to standard DNA barcodes, as revealed by Ji et al. [29] and Fu et al. [3]. Likewise, our barcoding analyses, conducted on various datasets using two different species-identification methods (tree-based and the distance-based), demonstrated that plastomes exhibited the highest species discriminatory power (90.47%). Furthermore, the plastome dataset revealed significantly higher species resolution than any other plastid DNA markers, including the standard plastid barcodes (*matK* + *rbcL* + *trnH-psbA*) and taxon-specific hypervariable DNA markers (Table 2). Additionally, nrDNA was found to be more preferable than ITS in our analyses (Tables 2 and 3). This highlights the importance of considering nrDNA in DNA barcoding studies.

The species resolution of both single plastid sequences and their combinations revealed low species resolution in *Acer*. Han et al. [39], Lin et al. [37], and Lin et al. [48] found that each single plastid locus (such as *matK*, *rbcL*, *trnH-psbA*, *trnL*-*trnF*, and *trnS*-*trnG*) provided a species resolution of less than 50% in *Acer*, due to the lack of genetic variations. Therefore, we constructed a concatenated dataset of standard plastid barcodes (*matK* + *rbcL* + *trnH*-*psbA*) to get more genetic variations. However, the species resolution of this dataset (61.90%) is still insufficient and is the lowest among all datasets (Table 2). Moreover, in this dataset (dataset E, Table 2), a total of 35 pairs of species exhibited 0 K2P distance, indicating a lack of interspecific variations and highlighting the challenge of DNA barcoding in *Acer*. The hypervariable regions in plastome were considered to be useful for species discrimination by Areces-Berazain et al. [57] and Dong et al. [52]. However, our results revealed that the two datasets with five hypervariable regions (dataset C and D; Table 2) showed significantly less resolution than that of the plastome dataset. Although *trnS-trnG* and *trnL*-*trnF* were previously used as taxon-specific markers in other studies [39, 60], our sliding window analysis did not support their designation as hypervariable regions in *Acer*.

ITS usually demonstrates a better performance than plastid DNA barcodes in most related studies [18] and *Acer* [37, 39]. Both Lin et al. [37] (73.09%) and our study revealed higher species resolution by ITS (66.67% in the tree-based method, and 71.43% in the distance-based method, respectively). However, ITS did not reveal interspecific variations for 9 pairs of species (0K2P^55^: 9, Table 2). Due to the longer sequence, nrDNA showed better performance (80.95% and 76.19% for the tree-based method and the distance-based method, respectively) than ITS.

### Signal underlying the improvement of species discrimination efficiency of barcodes

The increase in species resolution comes from additional interspecific variation [3]. In our study, the ITS dataset contains fewer variable characters than the *matK* + *rbcL* + *trnH-psbA* dataset (Table 1), however, it showed higher species resolution than the *matK* + *rbcL* + *trnH-psbA* dataset both in the tree-based and distance-based method (Table 2). The higher resolution of the ITS dataset may benefit from its richer interspecific variations because there were fewer species failed to be discriminated due to showing a minimum interspecific K2P distance of zero in the ITS dataset compared to the *matK* + *rbcL* + *trnH-psbA* dataset (3 vs. 7, Table 2). Our regression analysis did show a significantly negative correlation between the species resolution and the total number of 0K2P (Figure S7). This indicates that the lack of interspecific variations is a significant factor hindering the performance of DNA barcodes. Thus, investigating whether barcodes can provide sufficient interspecific variations before their use should be a priority.

Based on all 55 species sampled, we found substantially more species pairs with 0K2P distance in the *matK + rbcL + trnH-psbA* dataset (0K2P^55^: 35, Table 2), indicative of the lack of interspecific variations in this dataset. In contrast, the number of 0K2P species pairs in the plastome dataset is still zero, and plastomes were proved to have no shortage of interspecific variations because the range of minimum interspecific differences is 20 − 1,004, with an average of 220 (dataset A, Table 2). However, our undersampling of closely related species may lead to the current overestimation of interspecific variations in the plastome dataset.

Interspecific differences, which reflect the absolute number of interspecific variations, might be a more intuitive quantitative index than K2P distance. To eliminate the impact of undersampling of related species as much as possible, we downloaded some plastomes from NCBI to increase the sampled species to 128 (c. 81% of genus *Acer*) (Figure S6). We found plastomes can still provide abundant interspecific variations (Figure S8), with only 11 pairs of species exhibiting interspecific differences below 10, while 5 of them are subspecies pairs, and only one pair shows interspecific differences of zero (Table S4). It is worth noting that the potential hybridization may lead to underestimation of interspecific differences because hybridization could lead to the chloroplast capture between two species [3, 29, 34]. It follows that *Acer* plastomes could provide rich interspecific variations even in the case of underestimation.

### Potential reasons for species discrimination failure of plastome

The lack of variations between recently diversified species was regarded as one reason for species discrimination failure of barcodes 2.0 [3, 29, 34]. A negative correlation between the species discriminatory efficiency (SDE) of barcodes and the number of 0K2P was found in this study (Figure S7). However, when the number of 0K2P reaches zero, the SDE will not be improved even if the dataset continues to be longer and contains more variations. For instance, the two plastome-wide datasets (dataset A and B) get the same SDE (90.47%) in the tree-based method, though dataset A is longer and shows a significantly higher average of minimum interspecific difference (AMID) than dataset B (Table 2). This implies that the interspecific variation may have reached saturation for distinguishing existing species. Hybridization and/or incomplete lineage sorting (ILS) may be more possible causes limiting the further improvement of SDE, with a premise that the possibility of misidentification was ruled out because we have identified the specimen carefully and repeatedly. Nevertheless, our inadequate sampling of closely related species may have contributed to this inference.

*Acer* is a speciose genus with extensive interspecific hybridization under natural conditions [37–44, 46, 47]. Due to the characteristics of maternal inheritance of plastomes, hybridization can lead to the sharing of identical or similar plastomes (i.e., chloroplast capture) between species [3, 16, 22, 29, 61]. *Acer* plastomes are maternally inherited [62], they may thus not reflect species boundaries. For instance, *A. oliverianum* was 100% supported as monophyletic in our nrDNA ML tree (Fig. 3), however, the two individuals of this species were relatively distant in our plastome ML tree (Fig. 2). This cytonuclear conflict, accompanied by the grouping of *A. oliverianum* plastomes with other species reflects geographical proximity rather than taxonomic affinity (Fig. 2, Table S5), implying the presence of hybridization.

In addition to hybridization, ILS may be another cause of barcode failure, especially for recently differentiated species [34, 63, 64]. Previous studies reported that the formation of reciprocal monophyly alleles could take millions of years following the speciation event under different practical demographic parameters [65, 66]. For trees, reaching full monophyly may take 50 million years [67]. Therefore, though related *Acer* species have accomplished morphological differentiation, ancestral polymorphism at molecular levels may remain. For example, *A. coriaceifolium* was strongly resolved as monophyletic in our nrDNA ML tree and as a sister to *A. oblongum* (Fig. 3). However, one sample (FZ070) of *A. coriaceifolium* was found to cluster with *A. oblongum* in the plastome ML tree (Fig. 2). Given the taxonomic affinity between *A. coriaceifolium* and *A. oblongum* [42], ILS could not be excluded as a possible cause. More nuclear sequences are needed to confirm whether hybridization or ILS is responsible for this cytonuclear discordance.

### Suggestion for the usage of barcodes 2.0

Fu et al. [3] demonstrated that the concatenation of plastome and nrDNA can marginally improve the SDE in *Rhododendron*. Nevertheless, our result showed that the SDE was not enhanced when the plastome was combined with nrDNA (Table 2). Although combining them had increased the total number of variable sites (Table 1), the AMID of this dataset was lower than that of the plastome dataset (Table 2). This suggested that concatenating plastome and nrDNA had led to a reduction in the average minimum inter-species genetic variations available, which may be detrimental to species identification. Furthermore, the resulting ML tree inferred from the plastome + nrDNA dataset contained more polytomies than that of the plastome dataset (Fig. 2, Figure S3), illustrating the phylogenetic signal conflict between plastome and nrDNA. Given that the potential hybridization could blur inter-species genetic variations and what we mentioned above, combining plastome and nrDNA is not suggested for species identification in taxa with extensive hybridization similar to *Acer*.

We proved that plastomes can provide much richer interspecific variations and are therefore superior to standard barcodes and taxon-specific hypervariable plastid makers. However, due to the chloroplast capture resulting from hybridization [62], plastomes may not track species boundaries [16, 61]. Biparentally inherited nuclear sequences may be a better choice under this circumstance. For example, we found that two species that failed to be identified by plastomes were precisely successfully discriminated by nrDNA (Table 3). Given this outcome, nrDNA may compensate for the shortcomings of the plastome in species resolution when facing hybridization or ILS, and thus should be included in barcodes 2.0.

Notably, previous barcoding studies did not include ETS (external transcribed spacer) when using nrDNA (Figure S9), i.e., only used the 18 S–5.8 S-26 S cistron including ITS1 and 2 [3, 29, 34]. In our study, we additionally used a portion of ETS (with an aligned length of 834 bp), and this practice is conducive to improving the SDE (Table S6, Figure S10). We suggest incorporating the ETS sequence when using nrDNA in future studies.

Because of the significantly higher SDE of the barcodes 2.0 and the ever-decreasing cost of genome skimming, accompanied by the convenience of assembling plastomes and nrDNA, barcodes 2.0 will be a superior alternative compared to the combination of standard barcodes or any other plastid makers. However, for some more complex taxa, such as *Rhododendron* [3], *Fargesia* [33], and *Schima* [34], the SDE of barcodes 2.0 is unsatisfactory because lower than 60%. Hybridization, recent divergence, ILS, and taxonomic over-splitting are all suggested to be potential causes for the species discrimination failure of barcodes 2.0, and the addition of more nuclear sequences is recommended for these intractable genera [3, 29, 33, 34]. Nevertheless, not all taxa will be as complex as the above-mentioned genera. The situation of different genera still needs to be further studied, and there is still a lack of research on barcodes 2.0 so far.

### Insights into the phylogenetics of *Acer*

Previous studies on plastid phylogenetics mainly sampled only one species per Sects. [52, 56, 57], however, the phylogenetic position of a single species may not represent the systematic position of a given section if that section is non-monophyletic. Insufficient taxon sampling can lead to strong systematic bias [68], and the increase in taxon sampling can be highly conducive to improving phylogenetic analyses [69]. Thus, it is necessary to sample as many species as possible for a given section to confirm its plastid systematic position.

In our plastid phylogenetic analysis, we sampled over 80% of *Acer* species according to de Jong [35] (Fig. 4, Figure S5-S6). This contributed to confirming the plastid phylogenetic position of various sections. Notably, we found many prominent cytonuclear discordances between sections and within sections after comparing our plastid phylogeny with the phylogeny of Li et al. [49] based on 500 nuclear loci (Fig. 4). The causes of cytonuclear conflict include hybridization (especially organellar capture) and ILS [70–73]. ILS could apply to rapidly diverged species/lineages [74], i.e., for closely related species/lineages, which means that the affinity will be shown in both the plastid tree and nuclear tree, as revealed by Li et al. [73] in *Thuja*. However, most of the inter- and intra-section cytonuclear discordances illustrated in Fig. 4 merely reflect the closeness in one tree, while showing a quite distant relationship in another tree. ILS may not be the major factor accounting for these cytonuclear conflicts because the affinities were not shown in both the plastid tree and nuclear tree. And the most typical examples of this are the relationships between sects. *Platanoidea* and *Macrantha*, sects. *Arguta* and *Palmata*. It may follow that hybridizations are widely present between sections and have played a significant role in the evolution history of *Acer*. Nevertheless, to our knowledge, there is currently no research that details the extensive inter-section hybridization process of this genus. Further studies on gene flow using comprehensive nuclear genome-wide data and extensive species sampling are needed to explore this matter thoroughly in the future.

## Conclusion

Here we sequenced and assembled the plastomes as well as nrDNA of 83 individuals from 55 *Acer* species, and then assessed and compared the species discriminatory power of different barcoding datasets in *Acer*. Our results illustrated that both plastomes and nrDNA can effectively improve the species resolution in *Acer*, and plastomes exhibited the highest species resolution and most abundant interspecific variations. The use of nrDNA helps discriminate species that cannot be identified by plastomes. The plastid phylogenetic framework generated here enriched our understanding of the evolution of *Acer*, especially highlighting the role of hybridization in it.

## Methods

### Taxon sampling

83 individuals of 55 *Acer* species were sampled in this study (Table S5). Healthy leaves were collected and dried with silica gel. Voucher specimens were deposited at the herbarium of South China Botanical Garden (IBSC), Chinese Academy of Sciences, China. These 55 *Acer* species represent 13 major sections currently recognized in *Acer* [35, 42], 21 species were sampled with multiple (2–4) individuals, and the remaining 34 species with a single individual. All samples were identified by Dr. You-Sheng Chen. We also downloaded 184 *Acer* plastomes (Table S7) from GenBank. In total, 267 *Acer* plastomes (83 + 184) representing 128 species and 19 sections were used in our phylogenetic analysis and only sect. *Wardiana* (a monotypic section with only one species *A. wardii* W.W. Sm.) was not included, according to Xu et al. [42] and de Jong [35] (we adopted the treatment that sect. *Pentaphylla* was split into sect. *Oblonga* and *Pentaphylla* by Xu et al. [42]). In addition, the nrDNA (MW0702 and MW070204) and plastomes of two individuals, *Dimocarpus longan* and *Litchi chinensis*, were downloaded as outgroups (Table S7).

### DNA extraction, sequencing, assembly and annotation

Total genomic DNA was extracted from silica gel-dried leaves using the modified CTAB method [75]. Pair-end (PE) libraries with an average insert size of 270 base-pair (bp) were constructed at Beijing Genomics Institute (BGI, Shenzhen, China). Then, the libraries were sequenced on an Illumina X ten platform (San Diego, California) to generate 150 bp PE reads. Raw reads were subjected to quality check using FastQC (https://www.bioinformatics.babraham.ac.uk/projects/fastqc/). Clean reads were obtained after raw reads were trimmed and adaptors were removed by using Trimmomatric v0.36 [76]. Finally, each sample generated approximately 2–4 Gb of clean data. We assembled clean reads into plastome and nrDNA using the toolkit GetOrganelle v1.7.5 [77]. This toolkit extracts plastome reads and nuclear reads from total genomic reads for the following assembly by spades v3.10 [78]. As in rare cases, GetOrganelle generated some non-overlapping contigs instead of a complete plastome. Therefore, we mapped reads against these non-overlapping contigs to extend their ends to close the gap in Geneious, performing with medium-low sensitivity for 100 iterations.

Two independent approaches were applied to annotate 83 plastomes generated in this study. Firstly, the annotation of the plastome sequences was performed with GeSeq [79] choosing the plastome of *Acer miaotaiense* P. C. Tsoong (GenBank accession No.: NC_030343) as the reference genome. In the meantime, ARAGORN was selected as a third party to annotate tRNA. Secondly, we used MAFFT v7.388 [80] to align and annotate these plastome sequences by using the “Annotation Transfer” option with *Acer platanoides* L. (GenBank accession No.: MN864507) as reference in Geneious v2019.2.1. The annotation results from GeSeq and Geneious were subsequently compared and integrated. The annotation of nrDNA was conducted in Geneious with *Acer pentaphyllum* (GenBank accession number: MW070163) as the reference. The plastome map was drawn by using OGDRAW within GeSeq. Newly generated plastomes and nrDNA here were finally uploaded to GenBank (accession numbers in Table S8). Bwa v0.7.17-r1188 [81] and SAMtools v1.5 [82] were used to map the NGS data against corresponding plastome for validation of IR boundary, and the outputs were visualized in Geneious.

### Plastome analyses

The borders between the four plastome regions, i.e., LSC/IRb (JLB), SSC/IRb (JSB), SSC/IRa (JSA), and LSC/IRa (JLA), were visualized using the online program IRscope (https://irscope.shinyapps.io/irapp/). A sliding window analysis was performed in DnaSP v6.12.03 [83] to locate hypervariable genomic regions. The 83 *Acer* plastomes were aligned using MAFFT v7.388 [80] with default settings and used as the input file. The window length and step size were set to 600 bp and 100 bp, respectively. Those genomic regions with crest Pi (nucleotide diversity) values exceeding 0.020 and aligned lengths longer than 600 bp were identified as hypervariable genomic regions, and they were subsequently extracted from the plastome alignment using Geneious and analyzed separately to evaluate their characteristics. In addition, the analysis of indel polymorphism was also conducted in DnaSP.

### Data analyses for species discrimination

We constructed the following eight datasets based on our 83 samples of 55 *Acer* species: (A) the whole plastome with one IR removed, (B) the concatenation of the coding regions of protein-coding genes (PCG), rRNA genes and tRNA genes, (C) the combination of the four most variable markers identified by sliding window analysis in this study (*trnK*-*rps16* + *trnS*-*trnfM* + *ndhC*-*trnV* + *ndhF*-*trnL*), (D) *ycf1* (SSC portion), (E) the combination of three standard plastid barcodes (*matK* + *rbcL* + *trnH*-*psbA*) (F) the nrDNA sequence (ETS + 18 S + ITS1 + 5.8 S + ITS2 + 26 S), (G) ITS (ITS1 + 5.8 S + ITS2), (H) the combination of plastome and nrDNA.

All the coding sequences in annotated plastomes, including the coding sequences of protein, rRNA, and tRNA, were individually extracted by applying a Python script (https://github.com/Kinggerm/PersonalUtilities/blob/master/get_annotated_regions_from_gb.py). The ITS sequences were extracted from the annotated nrDNA assemblies in Geneious. For each dataset, the alignment was generated by MAFFT v7.388 [80] and then checked and manually modified in Geneious.

We accessed the species resolution of the above datasets using tree-based and distance-based methods. In the tree-based method, phylogenetic analyses were performed using maximum likelihood (ML) analysis in RAxML v8.2.12 [84] with GTR + Γ model, and 1,000 rapid bootstrap replicates were generated to evaluate the support values for each node. In the distance-based method, the pairwise distance was calculated using the Kimura 2-parameter (K2P) model [85] in the software MEGA7 [86]. The scatter plot of the minimum interspecific distance versus maximum intraspecific distance was generated to illustrate the barcoding gaps for each dataset. For comparing the richness of interspecific variations among different datasets, the pairwise differences (use No. of differences as a model when calculating pairwise distance) were also estimated in MEGA7.

In addition, a dataset containing 267 *Acer* plastomes (184 downloaded and 83 generated in this study) representing 128 species was constructed, and the ML analysis was performed on this dataset. Based on the resulting ML tree, 128 representative individuals (one individual per species) were selected for calculating interspecific differences and the following phylogenetic analysis. When situations where individuals of species from different sections nest with each other occur, our sampling principle is as follows: (1) retain the monophyletic and only-one-sample species; (2) prioritize our own samples; (3) retain individuals within their correct section while excluding those strays. This approach aims to mitigate potential identification errors and the impacts of hybridization, thus focusing more on inter-section relationships.

### Phylogenetic analysis

In total, 128 plastomes representing 128 *Acer* species (c. 81% of this genus) and 19 (95%) sections were sampled for the phylogenetic reconstruction. The 80 protein-coding sequences (CDSs) in annotated plastomes were individually extracted applying the aforesaid Python script and aligned using MAFFT with default settings. Two datasets were constructed based on these 80 CDSs using two partitioning strategies. For the first dataset, the alignments of the 80 CDSs were concatenated and regarded as a whole (i.e., unpartitioned strategy). For the second one, the alignments of the 80 CDSs were concatenated but partitioned (i.e., partitioned strategy). The ML and Bayesian inference (BI) analyses were both performed on these two datasets.

PartitionFinder2 [87] was used to select the best partitioning scheme and best-fit substitution models for the partitioned dataset. The model of evolution was set as ‘all’ and other parameters were kept as default. The 80 data blocks were consolidated into 31 subsets in the best-fit scheme (Table S9). These subsets and their corresponding substitution models were specified in both ML and BI analyses. For the unpartitioned dataset, GTR + I + G was selected as the best-fit substitution model using ModelTest-NG [88] under the corrected Akaike Information Criterion (AICc).

All ML analyses were performed using IQ-TREE [89] with 1000 ultrafast bootstraps [90]. All BI analyses were conducted in MrBayes v3.2.6 [91], and two MCMC runs were performed with 5 million generations and four chains, sampling every 1000 generations and discarding the 25% as burnin. LogCombiner within Beast v2.6.4 [92] was then applied to combine log files of the two MCMC runs. Tracer v1.7.2 [93] was finally used to confirm that the effective sample size (ESS) for each parameter was larger than 200 to ensure the convergence of MCMC run.

### Electronic supplementary material

Below is the link to the electronic supplementary material.

Supplementary Material 1

## Data Availability

All complete plastomes and nrDNA sequences used in this study are available from the National Center for Biotechnology Information (NCBI) (see Table S7, S8)  and the Science Data Bank at 10.57760/sciencedb.18484.
