# Supplementary material for: Testing plastomes and nuclear ribosomal DNA sequences as the next-generation DNA barcodes for species identification and phylogenetic analysis in Acer
Source: BMC Plant Biol. 2024 May 23;24:445. doi: 10.1186/s12870-024-05073-w (PMC11112886; doi:10.1186/s12870-024-05073-w)
Supplement: Supplementary file 1 — Supplementary Material 1 [file 12870_2024_5073_MOESM1_ESM.pdf]

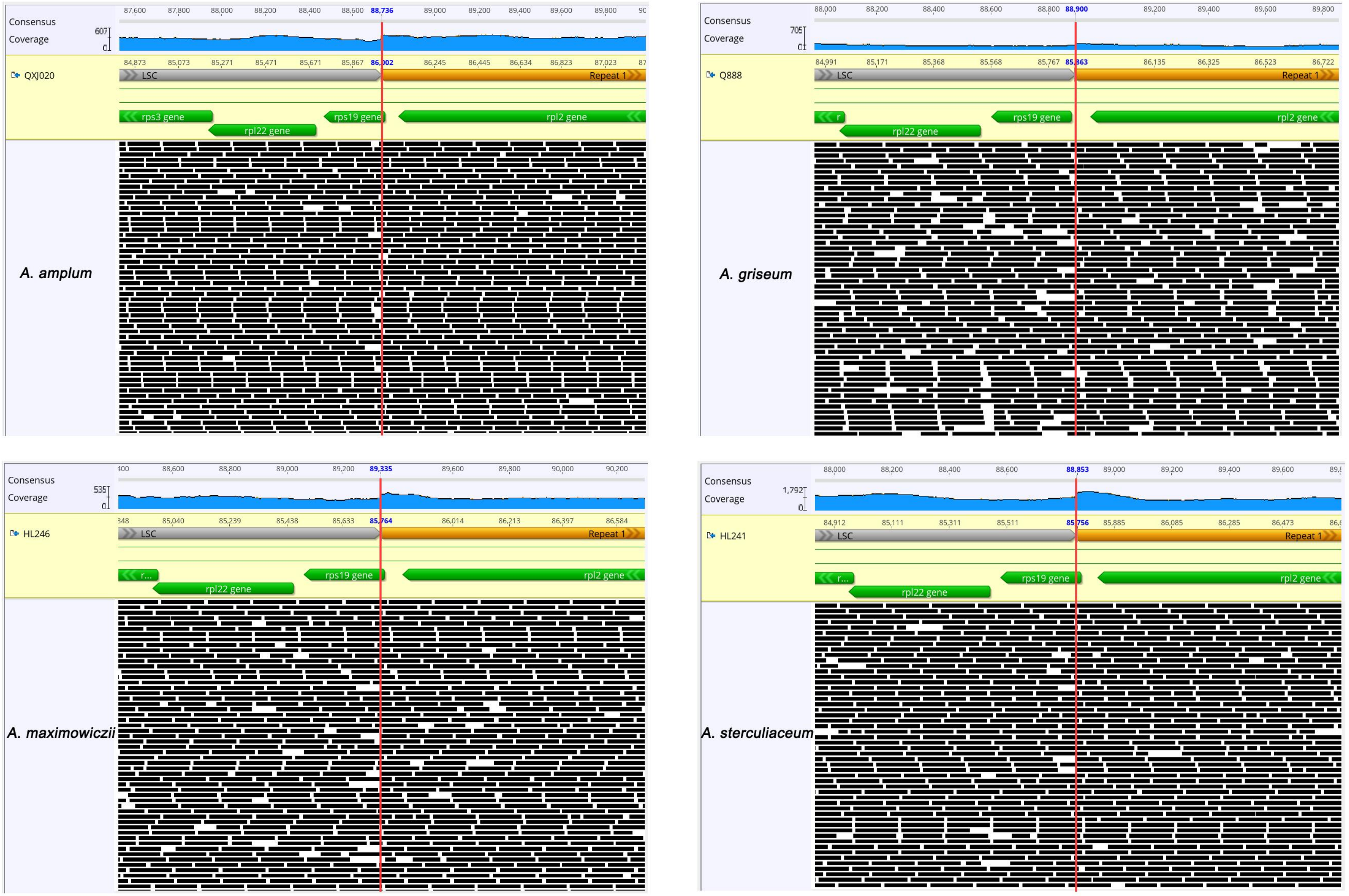

Fig. S1. The validations of IR boundary for 4 *Acer* species. The black bars represent NGS reads successfully mapped to the plastome, and the red vertical line marks the position of the IR boundary. The image was captured from Geneious.

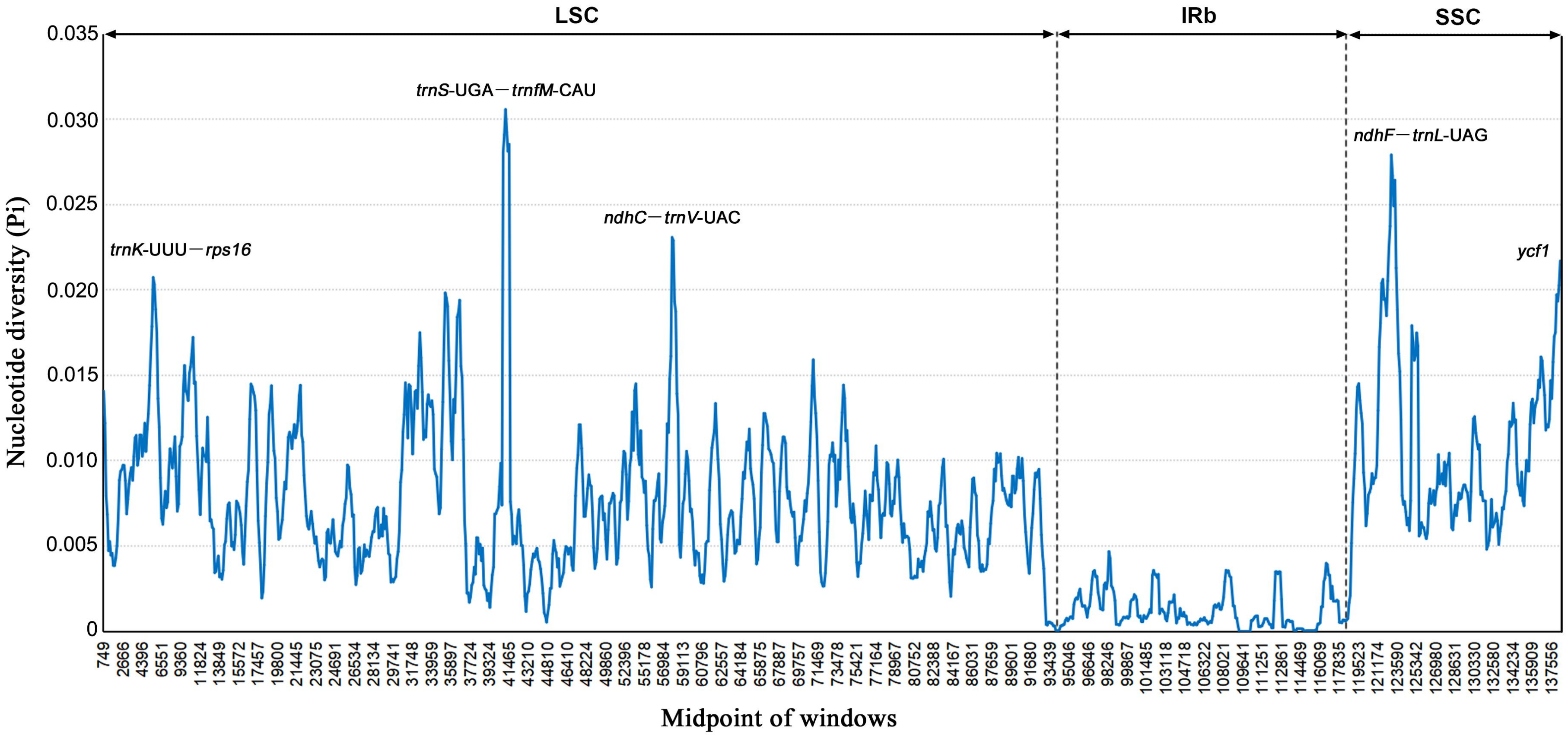

Fig. S2. Sliding window analysis of *Acer* plastomes. window length: 600 bp, step size: 100 bp.

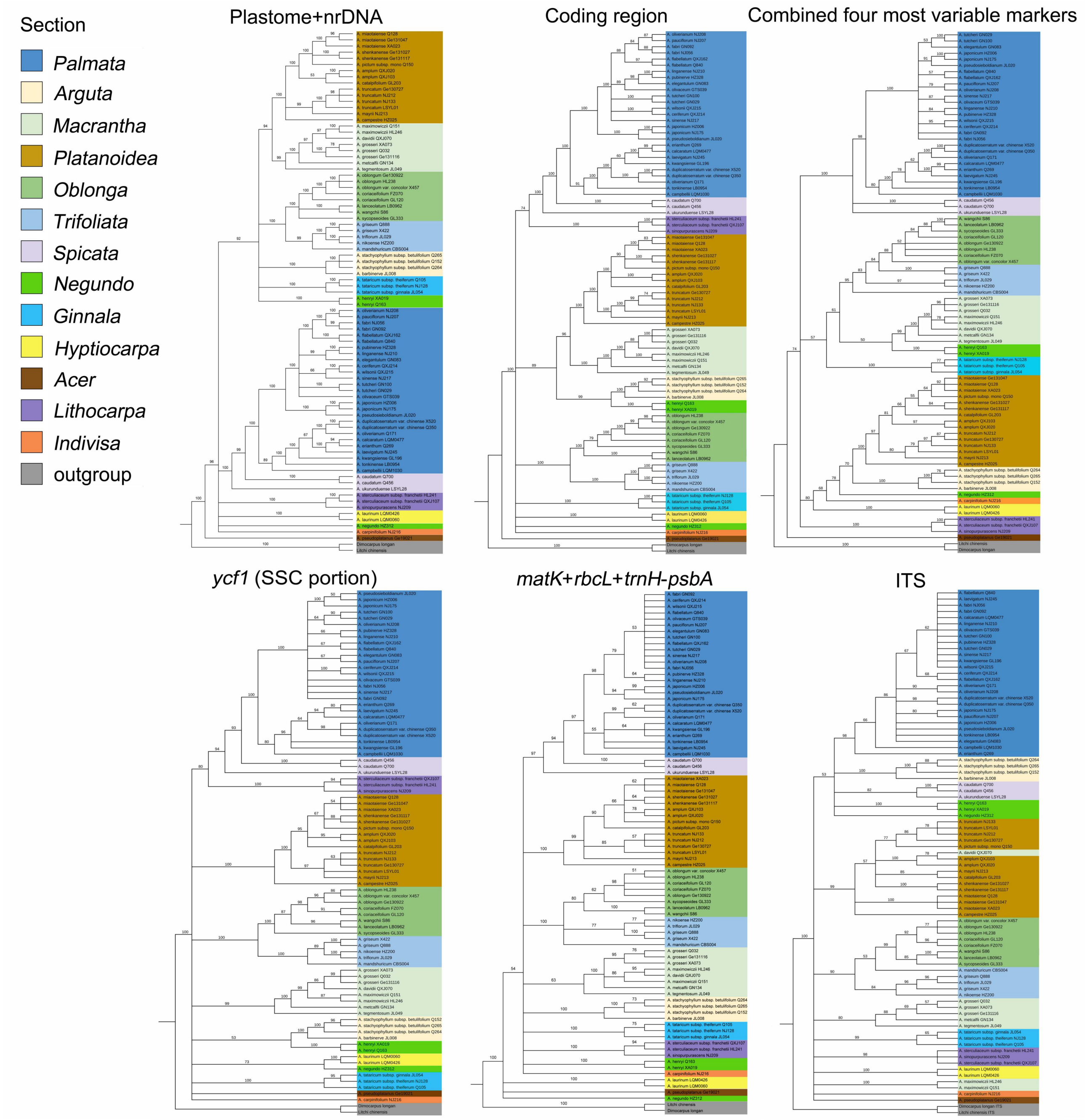

Fig. S3. ML trees inferred from different barcoding datasets. ML bootstrap support (BS) values are shown at nodes. Clades were set to polytomy when BS<50%.

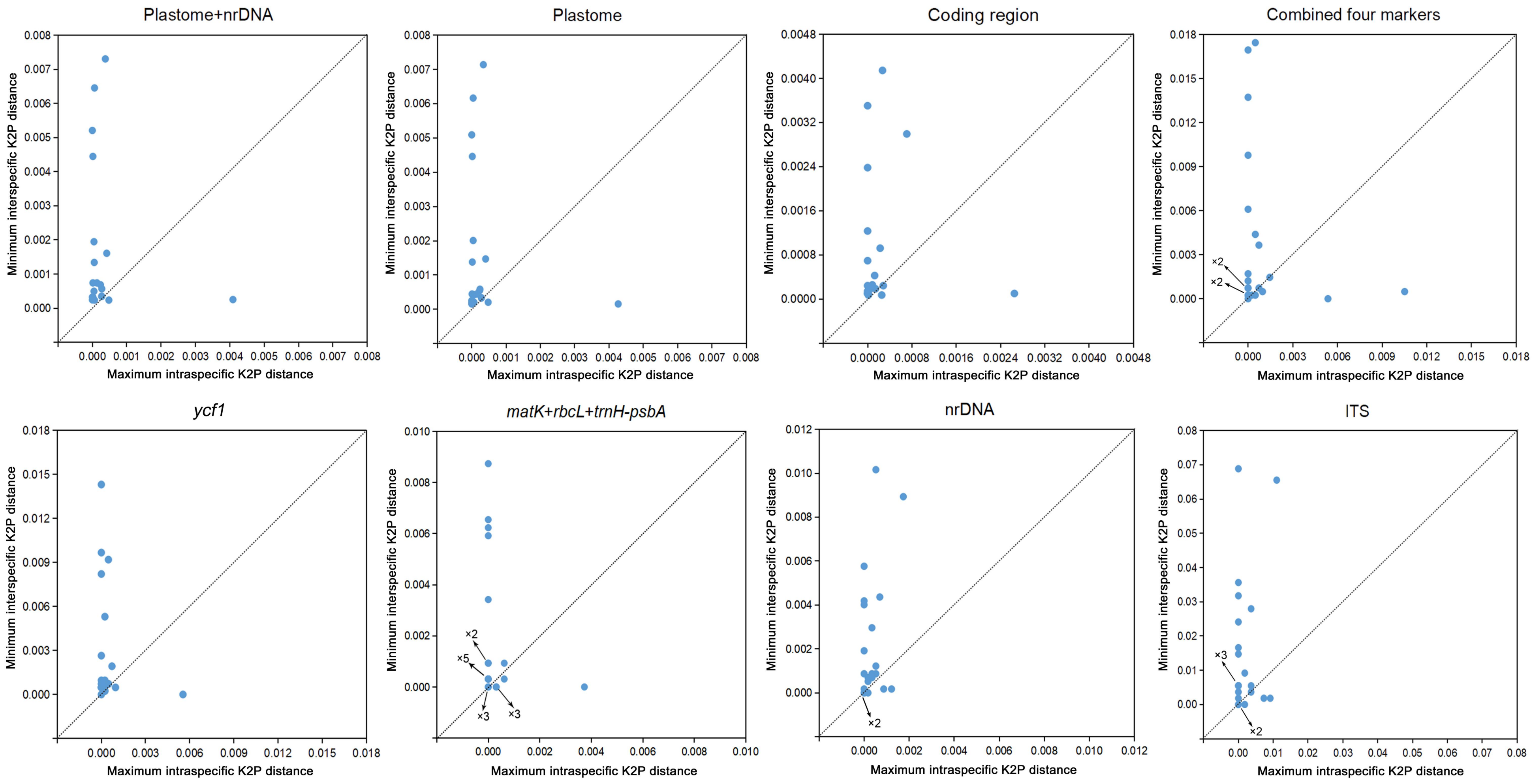

Fig. S4. Barcoding gaps of various datasets.

Fig. S5. ML tree inferred from the dataset comprised of 267 *Acer* plastomes. Clades were set to polytomy when BS<50%. The individuals of non-monophyletic species were marked with red dots at the end of the branches. Accessions that used for the final phylogenetic analysis were underlined in red.

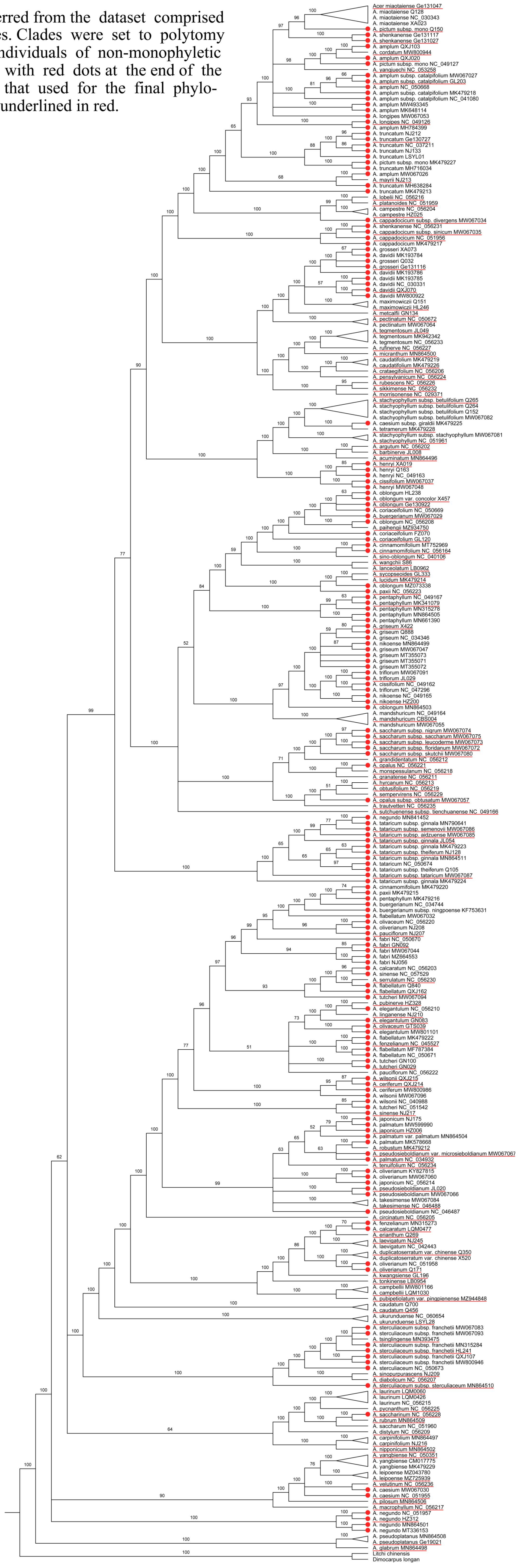

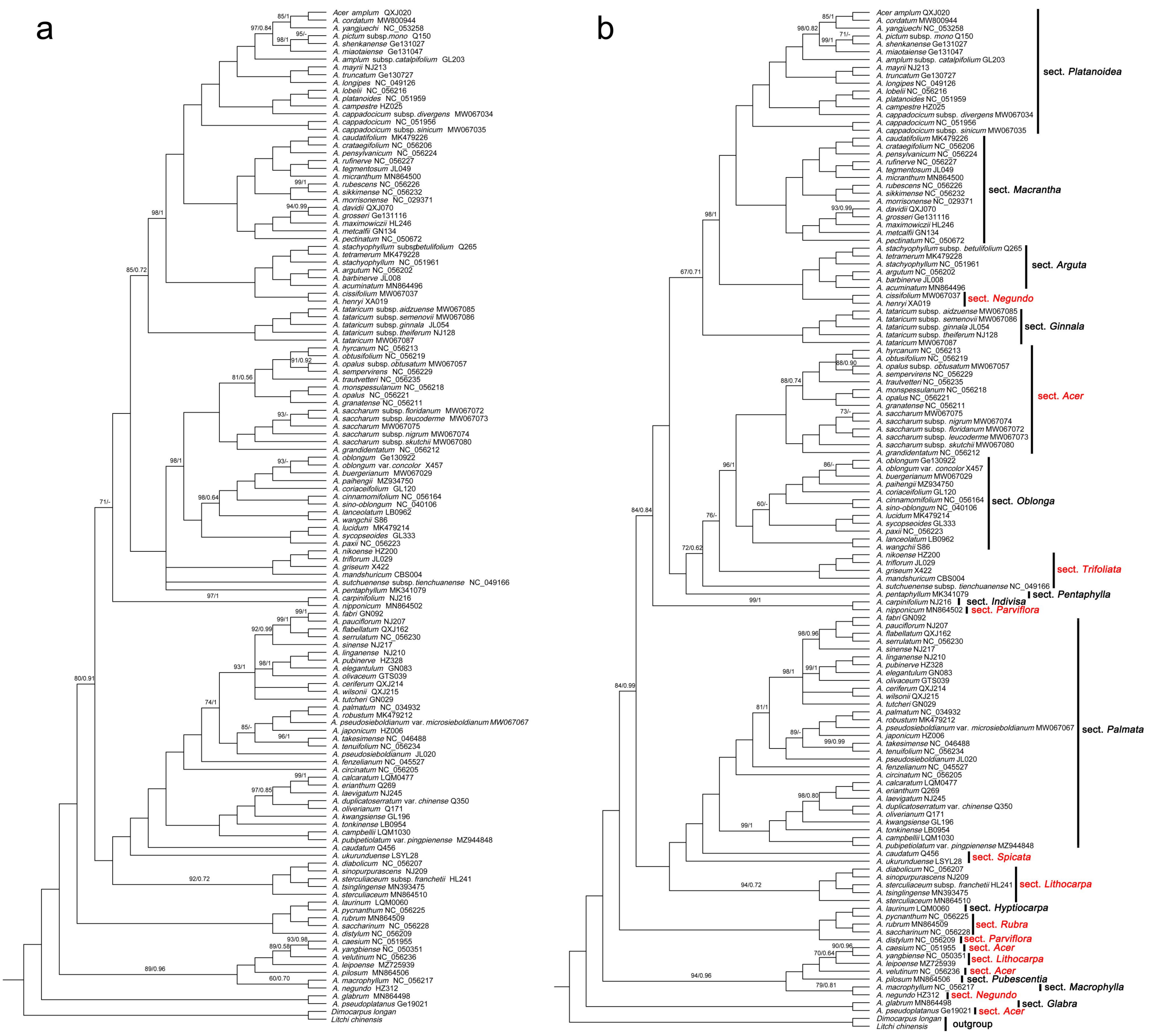

Fig. S6. *Acer* phylogenies inferred from partitioned (a) and unpartitioned (b) 80 CDSs dataset. ML bootstrap (BS) values from IQ-TREE and the posterior probabilities (PP) calculated from MrBayes are shown at nodes, except nodes with 100% BS and 1.0 PP, '-'indicates PP under 0.5. Clades were set to polytomy when BS<50%. Non-monophyletic sections are shown in red.

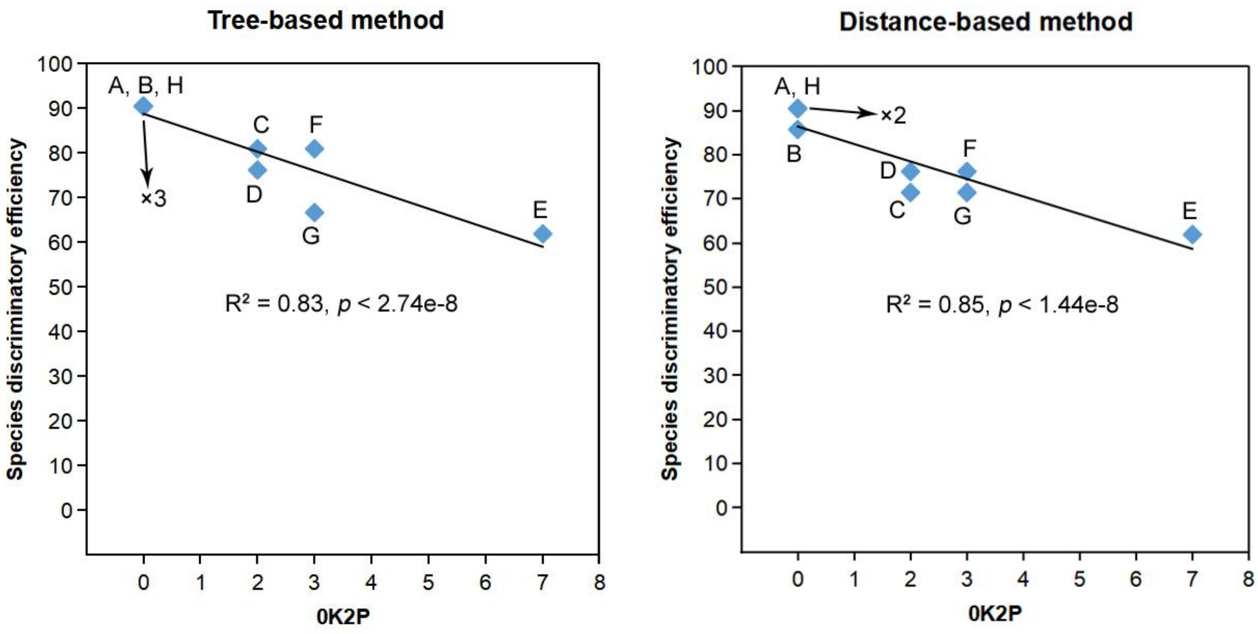

Fig. S7. Regression analysis between the species discriminatory efficiency and the 0K2P. 0K2P: the total number of species (with multiple individuals) that failed to be discriminated due to showing minimum interspecific K2P distance of zero. The dataset codes are shown beside the dots.

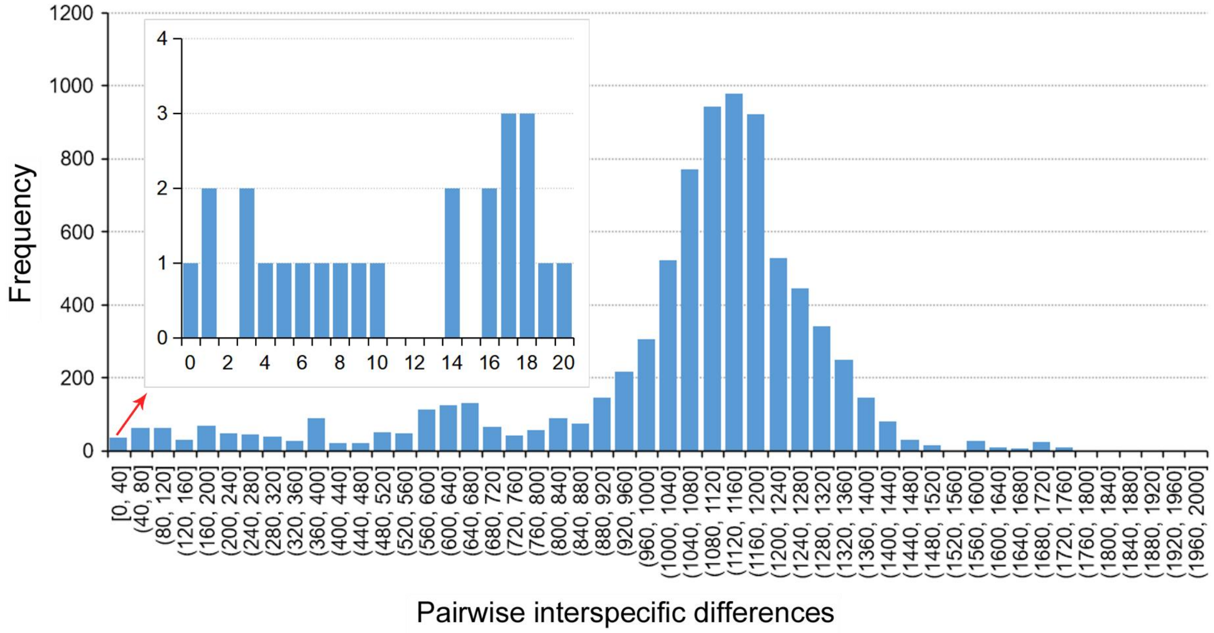

Fig. S8. Histogram of the frequency of pairwise interspecific differences for the dataset comprised of 128 *Acer* plastomes (species).

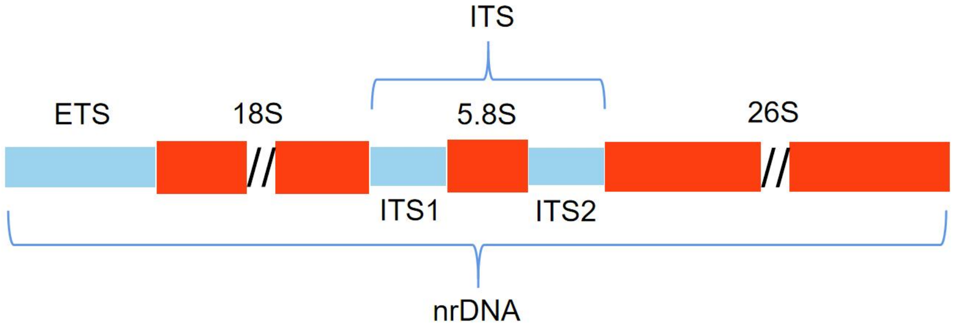

nrDNA: nuclear ribosomal DNA;
ITS: internal transcribed spacer;
ETS: external transcribed spacer;

Fig. S9. The structure of nrDNA used in this study.

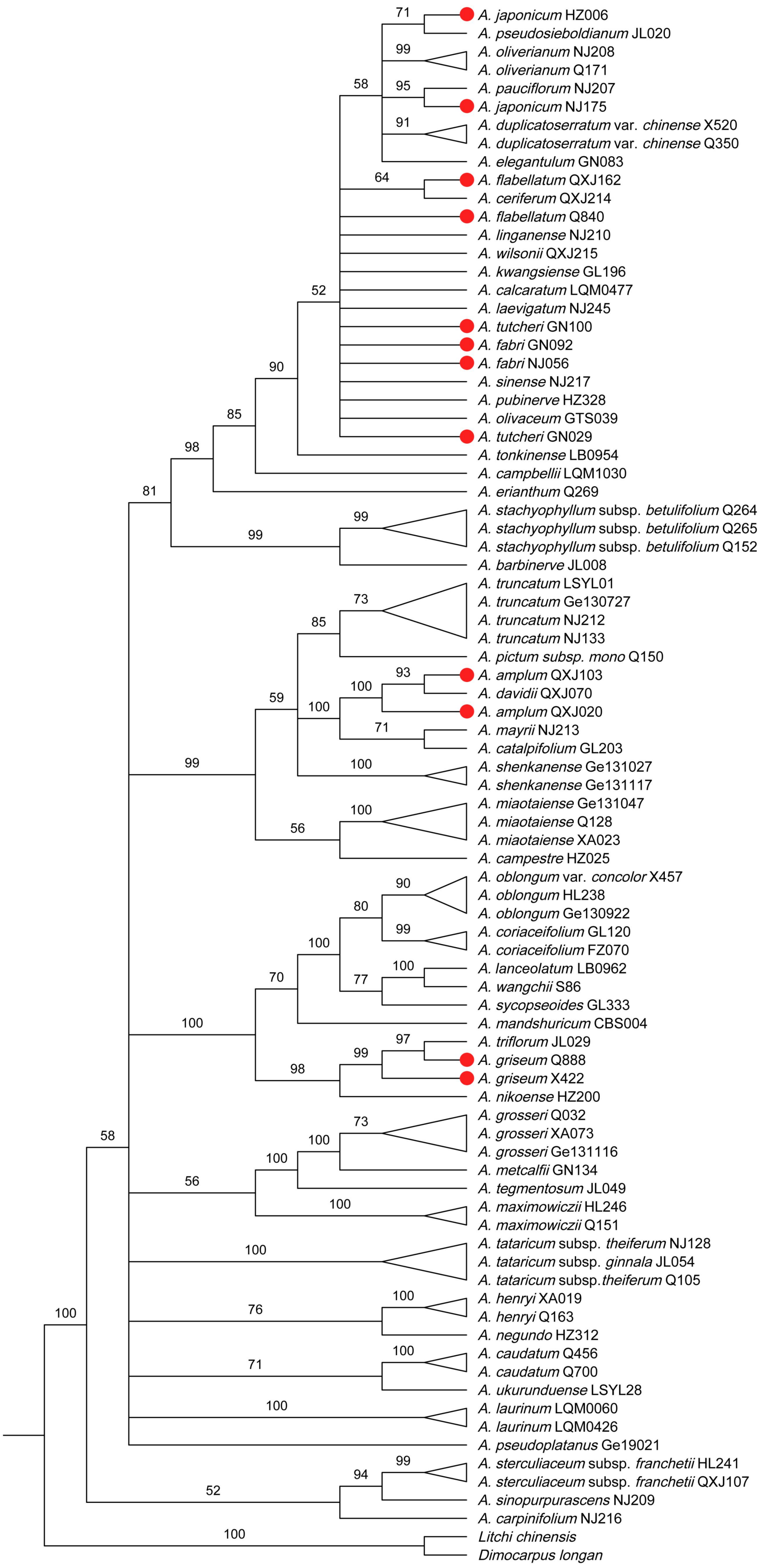

Fig. S10. ML tree inferred from the nrDNA dataset without ETS. Clades were set to polytomy when BS<50%. The individuals of non-monophyletic species were marked with red dots at the end of the branches. ETS: external transcribed spacer.

Table S1. Major characteristics of *Acer* plastomes generated in this study.

Species	Section	DNA accession	Size (bp)	LSC(bp)	SSC(bp)	IR(bp)	GC%				Number of genes	PCG	tRNA	rRNA
							Total	LSC	SSC	IR				
Acer amplum	*Platanoidea*	QXJ020	156,252	86,001	18,067	26,092	37.9	36.1	32.2	42.9	138	89	40	8
A. amplum	*Platanoidea*	QXJ103	156,247	85,996	18,067	26,092	37.9	36.1	32.2	42.9	138	89	40	8
A. barbinerve	*Arguta*	JL008	155,985	85,774	18,057	26,077	37.9	36.0	32.1	42.9	138	89	40	8
A. calcaratum	*Palmata*	LQM0477	156,659	85,048	18,091	26,760	37.9	36.0	32.2	42.7	139	90	40	8
A. campbellii	*Palmata*	LQM1030	156,815	85,204	18,125	26,743	37.9	36.1	32.1	42.8	139	90	40	8
A. campestre	*Platanoidea*	HZ025	156,314	86,009	18,107	26,099	37.9	36.0	32.1	42.9	138	89	40	8
A. carpinifolium	*Indivisa*	NJ216	155,568	85,449	18,079	26,020	38.0	36.2	32.4	43.0	138	89	40	8
A. catalpifolium	*Platanoidea*	GL203	156,424	86,178	18,066	26,090	37.9	36.0	32.2	42.9	138	89	40	8
A. caudatum	*Spicata*	Q456	156,262	84,655	18,115	26,746	37.9	36.1	32.2	42.8	139	90	40	8
A. caudatum	*Spicata*	Q700	156,244	84,626	18,114	26,752	37.9	36.1	32.2	42.8	139	90	40	8
A. ceriferum	*Palmata*	QXJ214	157,029	85,356	18,167	26,753	37.9	36.0	32.3	42.8	139	90	40	8
A. coriaceifolium	*Oblonga*	FZ070	156,034	85,839	18,065	26,065	38.0	36.1	32.3	42.9	138	89	40	8
A. coriaceifolium	*Oblonga*	GL120	156,089	85,827	18,118	26,072	37.9	36.1	32.3	42.9	138	89	40	8
A. davidii	*Macrantha*	QXJ070	155,896	85,723	17,983	26,095	38.0	36.2	32.3	42.9	138	89	40	8
A. duplicatoserratum var. *chinense*	*Palmata*	Q350	156,920	85,269	18,171	26,740	37.9	36.0	32.1	42.8	139	90	40	8
A. duplicatoserratum var. *chinense*	*Palmata*	X520	156,857	85,274	18,103	26,740	37.9	36.0	32.1	42.8	139	90	40	8
A. elegantulum	*Palmata*	GN083	156,896	85,262	18,126	26,754	37.9	36.0	32.2	42.7	139	90	40	8
A. erianthum	*Palmata*	Q269	156,571	84,984	18,085	26,751	37.9	36.1	32.2	42.8	139	90	40	8
A. fabri	*Palmata*	GN092	156,919	85,296	18,113	26,755	37.9	36.0	32.2	42.7	139	90	40	8
A. fabri	*Palmata*	NJ056	156,815	85,389	18,102	26,662	37.9	36.0	32.2	42.8	139	90	40	8
A. flabellatum	*Palmata*	Q840	156,962	85,334	18,122	26,753	37.9	36.0	32.2	42.8	139	90	40	8
A. flabellatum	*Palmata*	QXJ162	156,961	85,297	18,158	26,753	37.9	36.0	32.2	42.8	139	90	40	8
A. griseum	*Trifoliata*	Q888	156,079	85,862	18,147	26,035	37.9	36.1	32.3	42.9	138	89	40	8
A. griseum	*Trifoliata*	X422	156,079	85,862	18,147	26,035	37.9	36.1	32.3	42.9	138	89	40	8
A. grosseri	*Macrantha*	Ge131116	155,904	85,736	17,982	26,093	38.0	36.2	32.3	42.9	138	89	40	8
A. grosseri	*Macrantha*	Q032	155,904	85,736	17,982	26,093	38.0	36.2	32.3	42.9	138	89	40	8
A. grosseri	*Macrantha*	XA073	155,897	85,729	17,982	26,093	38.0	36.2	32.3	42.9	138	89	40	8
A. henryi	*Negundo*	Q163	156,328	86,061	18,071	26,098	37.9	36.0	32.2	42.9	138	89	40	8
A. henryi	*Negundo*	XA019	156,352	86,060	18,096	26,098	37.9	36.0	32.2	42.9	138	89	40	8
A. japonicum	*Palmata*	NJ175	156,958	85,306	18,140	26,756	37.9	36.0	32.2	42.8	139	90	40	8
A. japonicum	*Palmata*	HZ006	157,017	85,363	18,142	26,756	37.9	36.0	32.2	42.8	139	90	40	8

Table S1. (continued)

Species	Section	DNA accession	Size (bp)	LSC(bp)	SSC(bp)	IR(bp)	GC%				Number of genes	PCG	tRNA	rRNA
							Total	LSC	SSC	IR				
A. kwangnanense	*Oblonga*	LB0962	155,727	85,541	18,074	26,056	38.0	36.2	32.3	42.9	138	89	40	8
A. kwangsiense	*Palmata*	GL196	156,789	85,234	18,077	26,739	37.9	36.0	32.2	42.8	139	90	40	8
A. laevigatum	*Palmata*	NJ245	156,552	84,957	18,115	26,740	37.9	36.1	32.2	42.8	139	90	40	8
A. laurinum	*Hyptiocarpa*	LQM0060	156,004	85,786	18,052	26,083	37.9	36.0	32.2	42.9	138	89	40	8
A. laurinum	*Hyptiocarpa*	LQM0426	156,013	85,795	18,052	26,083	37.9	36.0	32.2	42.9	138	89	40	8
A. linganense	*Palmata*	NJ210	156,918	85,287	18,123	26,754	37.9	36.0	32.2	42.7	139	90	40	8
A. mandshuricum	*Trifoliata*	CBS004	156,250	86,058	18,058	26,067	37.9	36.0	32.3	42.9	138	89	40	8
A. maximowiczii	*Macrantha*	HL246	156,105	85,763	17,962	26,190	38.0	36.2	32.3	43.0	140	89	42	8
A. maximowiczii	*Macrantha*	Q151	155,986	85,798	17,988	26,100	38.0	36.2	32.3	42.9	138	89	40	8
A. mayrii	*Platanoidea*	NJ213	156,238	85,991	18,067	26,090	37.9	36.1	32.2	42.9	138	89	40	8
A. metcalfii	*Macrantha*	GN134	155,769	85,618	17,983	26,084	38.0	36.2	32.3	42.9	138	89	40	8
A. miaotaiense	*Platanoidea*	Ge131047	156,243	85,991	18,068	26,092	37.9	36.1	32.2	42.9	138	89	40	8
A. miaotaiense	*Platanoidea*	Q128	156,243	85,991	18,068	26,092	37.9	36.1	32.2	42.9	138	89	40	8
A. miaotaiense	*Platanoidea*	XA023	156,241	85,989	18,068	26,092	37.9	36.1	32.2	42.9	138	89	40	8
A. negundo	*Negundo*	HZ312	155,916	85,657	18,091	26,084	37.9	36.1	32.3	42.9	138	89	40	8
A. nikoense	*Trifoliata*	HZ200	156,096	85,888	18,050	26,079	37.9	36.0	32.4	42.9	138	89	40	8
A. oblongum	*Oblonga*	Ge130922	155,947	85,777	18,042	26,064	38.0	36.1	32.3	42.9	138	89	40	8
A. oblongum	*Oblonga*	HL238	155,959	85,789	18,042	26,064	38.0	36.1	32.3	42.9	138	89	40	8
A. oblongum var. *concolor*	*Oblonga*	X457	155,915	85,744	18,043	26,064	38.0	36.1	32.3	42.9	138	89	40	8
A. olivaceum	*Palmata*	GTS039	156,716	85,061	18,149	26,753	37.9	36.0	32.2	42.8	139	90	40	8
A. oliverianum	*Palmata*	NJ208	156,799	85,191	18,098	26,755	37.9	36.0	32.2	42.7	139	90	40	8
A. oliverianum	*Palmata*	Q171	156,890	85,275	18,135	26,740	37.9	36.0	32.1	42.8	139	90	40	8
A. pauciflorum	*Palmata*	NJ207	157,008	85,397	18,107	26,752	37.9	36.0	32.2	42.8	139	90	40	8
A. pictum subsp. *mono*	*Platanoidea*	Q150	156,247	85,967	18,094	26,093	37.9	36.1	32.2	42.9	138	89	40	8
A. pseudoplatanus	*Acer*	Ge19021	155,816	85,690	17,960	26,083	37.9	36.1	32.2	42.9	138	89	40	8
A. pseudosieboldianum	*Palmata*	JL020	157,014	85,391	18,143	26,740	37.9	36.0	32.2	42.8	139	90	40	8
A. pubinerve	*Palmata*	HZ328	156,869	85,239	18,120	26,755	37.9	36.0	32.2	42.7	139	90	40	8
A. shenkanense	*Platanoidea*	Ge131027	156,163	85,923	18,058	26,091	37.9	36.1	32.2	42.9	138	89	40	8
A. shenkanense	*Platanoidea*	Ge131117	156,167	85,924	18,059	26,092	37.9	36.1	32.2	42.9	138	89	40	8
A. sinense	*Palmata*	NJ217	156,949	85,338	18,105	26,753	37.9	36.0	32.2	42.8	139	90	40	8
A. sinopurpurascens	*Lithocarpa*	NJ209	156,059	85,797	18,110	26,076	38.0	36.2	32.4	43.0	138	89	40	8
A. stachyophyllum subsp. *betulifolium*	*Arguta*	Q152	156,118	85,860	18,096	26,081	37.9	36.0	32.1	42.9	138	89	40	8
A. stachyophyllum subsp. *betulifolium*	*Arguta*	Q264	155,980	85,747	18,065	26,084	37.9	36.0	32.1	42.9	138	89	40	8
A. stachyophyllum subsp. *betulifolium*	*Arguta*	Q265	156,108	85,873	18,073	26,081	37.9	36.0	32.1	42.9	138	89	40	8

Table S1. (continued)

Species	Section	DNA accession	Size (bp)	LSC(bp)	SSC(bp)	IR(bp)	GC%				Number of genes	PCG	tRNA	rRNA
							Total	LSC	SSC	IR				
A. sterculiaceum subsp. *franchetii*	*Lithocarpa*	HL241	156,032	85,755	18,137	26,070	38.0	36.2	32.4	42.9	138	89	40	8
A. sterculiaceum subsp. *franchetii*	*Lithocarpa*	QXJ107	156,187	85,888	18,145	26,077	38.0	36.2	32.4	42.9	138	89	40	8
A. sycopseoides	*Oblonga*	GL333	156,101	85,875	18,090	26,068	38.0	36.1	32.3	42.9	138	89	40	8
A. tataricum subsp. *ginnala*	*Ginnala*	JL054	155,595	85,369	18,042	26,092	38.0	36.2	32.4	42.9	138	89	40	8
A. tataricum subsp. *theiferum*	*Ginnala*	NJ128	155,625	85,382	18,041	26,101	38.0	36.3	32.4	42.9	138	89	40	8
A. tataricum subsp. *theiferum*	*Ginnala*	Q105	155,602	85,354	18,052	26,098	38.0	36.3	32.4	42.9	138	89	40	8
A. tegmentosum	*Macrantha*	JL049	156,512	86,164	18,104	26,122	37.9	36.0	32.2	42.9	138	89	40	8
A. tonkinense	*Lithocarpa*	LB0954	156,743	85,254	18,047	26,721	37.9	36.0	32.2	42.8	139	90	40	8
A. triflorum	*Trifoliata*	JL029	155,808	85,597	18,053	26,079	37.9	36.0	32.3	42.9	138	89	40	8
A. truncatum	*Platanoidea*	Ge130727	156,261	86,014	18,071	26,088	37.9	36.0	32.2	42.9	138	89	40	8
A. truncatum	*Platanoidea*	LSYL01	156,225	85,980	18,069	26,088	37.9	36.1	32.2	42.9	138	89	40	8
A. truncatum	*Platanoidea*	NJ133	156,261	86,014	18,071	26,088	37.9	36.0	32.2	42.9	138	89	40	8
A. truncatum	*Platanoidea*	NJ212	156,261	86,014	18,071	26,088	37.9	36.0	32.2	42.9	138	89	40	8
A. tutcheri	*Palmata*	GN100	157,291	85,339	18,386	26,783	37.9	36.0	32.2	42.7	139	90	40	8
A. tutcheri	*Palmata*	GN029	157,267	85,339	18,362	26,783	37.9	36.0	32.2	42.7	139	90	40	8
A. ukurunduense	*Spicata*	LSYL28	156,539	85,060	18,009	26,735	37.9	36.1	32.2	42.8	139	90	40	8
A. wangchii	*Palmata*	S86	155,852	85,650	18,090	26,056	38.0	36.1	32.2	42.9	138	89	40	8
A. wilsonii	*Palmata*	QXJ215	157,002	85,329	18,167	26,753	37.9	36.0	32.3	42.8	139	90	40	8

Table S2. List of genes present in *Acer* plastomes generated in this study.

Group of gene	Genes name	Amount
Photosystem I	*psaA*, *psaB*, *psaC*, *psaI*, *psaJ*	5
Photosystem II	*psbA*, *psbB*, *psbC*, *psbD*, *psbE*, *psbF*, *psbH*, *psbI*, *psbJ*, *psbK*, *psbL*, *psbM*, *psbN*, *psbT*, *psbZ*	15
Photosystem assembly factors	*ycf3***, *ycf4*	2
Cytochrome b/f complex	*petA*, *petB**, *petD**, *petG*, *petL*, *petN*	6
ATP synthase complex	*atpA*, *atpB*, *atpE*, *atpF**, *atpH*, *atpI*	6
NADH dehydrogenase complex	*ndhA**, *ndhB** (×2), *ndhC*, *ndhD*, *ndhE*, *ndhF*, *ndhG*, *ndhH*, *ndhI*, *ndhJ*, *ndhK*	12
Large subunit of RuBisCO	*rbcL*	1
Maturase	*matK*	1
RNA polymerase subunits	*rpoA*, *rpoB*, *rpoC1**, *rpoC2*	4
Small subunit ribosomal proteins	*rps2*, *rps3*, *rps4*, *rps7* (×2), *rps8*, *rps11*, *rps12** (×2), *rps14*, *rps15*, *rps16**, *rps18*, *rps19* (×2 in sect. *Palmata* and sect. *Spicata*)	14 (15)
Large subunit ribosomal proteins	*rpl2** (×2), *rpl14*, *rpl16**, *rpl20*, *rpl22*, *rpl23* (×2), *rpl32*, *rpl33*, *rpl36*	11
Subunit of acetyl-CoA-carboxylase	*accD*	1
Subunit of Clp-protease	*clpP***	1
Translation initiation factor	*infA* Ψ	1
Inner envelope membrane protein	*cemA*	1
Cytochrome c biogenesis protein	*ccsA*	1
Genes of unknown function	*orf42* (×2), *ycf1*, *ycf1*a, *ycf2* (×2), *ycf15* (×2)	8
Ribosomal RNAs	*rrn4.5* (×2), *rrn5* (×2), *rrn16* (×2), *rrn23* (×2)	8
Transfer RNAs	*trnA-UGC** (×2), *trnC-GCA*, *trnD-GUC*, *trnE-UUC*, *trnF-GAA*, *trnfM-CAU*, *trnG-GCC*, *trnG-UCC**, *trnH-GUG*, *trnI-CAU* (×2), *trnI-GAU** (×2), *trnK-UUU**, *trnL-CAA* (×2), *trnL-UAA**, *trnL-UAG*, *trnM-CAU* (×2), *trnN-GUU* (×2), *trnP-GGG*, *trnP-UGG*, *trnQ-UUG*, *trnR-ACG* (×2) (×4 in *A. maximowiczii* HL246), *trnR-UCU*, *trnS-GCU*, *trnS-GGA*, *trnS-UGA*, *trnT-GGU* (×2), *trnT-UGU*, *trnV-GAC* (×2), *trnV-UAC**, *trnW-CCA*, *trnY-GUA*	40 (42)
Total		138 (139/140)

Note: Genes marked with one asterisk (*) contain one intron; two asterisk (*) represent double introns. *Ycf1a* is the fragment of *ycf1*.

Table S3. Characteristic statistics of five hypervariable regions.

Regions	Aligned length (bp)	Variable sites		PI sites		Pi	Haplotypes	No. of indels
		Number	%	Number	%			
trnK-UUU—*rps16*	1,256	132	10.51	90	7.17	0.01933	43	97
trnS-UGA—*trnfM*-CAU	1,675	136	8.12	92	5.49	0.01889	44	71
ndhC—*trnV*-UAC	1,040	79	7.60	60	5.77	0.02339	35	62
ndhF—*trnL*-UAG	2,540	282	11.10	191	7.52	0.02265	58	170
ycf1 (SSC portion)	4,359	513	11.77	291	6.68	0.01331	63	24

Note: Pi: Nucleotide diversity; PI sites: Parsimony informative sites

Table S4. Species pairs that with interspecific differences below 10.

Species 1	Species 2	Difference
Acer saccharum subsp. *floridanum* MW067072	*A. saccharum* subsp. *leucoderme* MW067073	0
A. tataricum subsp. *aidzuense* MW067085	*A. tataricum* subsp. *semenovii* MW067086	1
A. argutum NC_056202	*A. barbinerve* JL008	1
A. sterculiaceum subsp. *franchetii* HL241	*A. tsinglingense* MN393475	3
A. ceriferum QXJ214	*A. wilsonii* QXJ215	3
A. tataricum subsp. *ginnala* JL054	*A. tataricum* subsp. *semenovii* MW067086	4
A. tataricum subsp. *aidzuense* MW067085	*A. tataricum* subsp. *ginnala* JL054	5
A. monspessulanum NC_056218	*A. opalus* NC_056221	6
A. amplum QXJ020	*A. cordatum* MW800944	7
A. rubescens NC_056226	*A. sikkimense* NC_056232	8
A. oblongum Ge130922	*A. oblongum var. concolor* X457	9

Table S5. Collection information of *Acer* plant materials.

Species	Section	Locality	Sample ID	Voucher specimen	Collector
Acer pseudoplatanus L.	*Acer*	Ringve Bot. Garden, Trondreim, Norway	Ge19021	Ge19021	X.J. Ge
A. barbinerve Maxim. ex Miq.	*Arguta*	Changbai mountain, Jilin, China	JL008	CBS018	J. Ye
A. stachyophyllum subsp. betulifolium (Maxim.) P.C. de Jong	*Arguta*	Baoji, Shaanxi, China	Q152	Q152	S.L. Dong
A. stachyophyllum subsp. betulifolium (Maxim.) P.C. de Jong	*Arguta*	Baoji, Shaanxi, China	Q264	Q264	S.L. Dong
A. stachyophyllum subsp. betulifolium (Maxim.) P.C. de Jong	*Arguta*	Baoji, Shaanxi, China	Q265	Q265	S.L. Dong
A. tataricum subsp. ginnala (Maxim.) Wesm.	*Ginnala*	Changbai mountain, Jilin, China	JL054	CBS008	J. Ye
A. tataricum subsp. theiferum (W.P.Fang) Y.S. Chen & P.C. de Jong	*Ginnala*	Zhongshan Bot. Garden, Nanjing, Jiangsu, China	NJ128	NJ128	Y. Xu et al.
A. tataricum subsp. theiferum (W.P.Fang) Y.S. Chen & P.C. de Jong	*Ginnala*	Baoji, Shaanxi, China	Q105	Q105	S.L. Dong
A. laurinum Hassk.	*Hyptiocarpa*	Xishuangbanna, Yunnan, China	LQM0060	BB1908-0217	Q.M. Li
A. laurinum Hassk.	*Hyptiocarpa*	Xishuangbanna, Yunnan, China	LQM0426	TD54-048	Q.M. Li
A. carpinifolium Siebold & Zucc.	*Indivisa*	Zhongshan Bot. Garden, Nanjing, Jiangsu, China	NJ216	NJ216	Y. Xu et al.
A. sterculiaceum subsp. franchetii (Pax) A.E. Murray	*Lithocarpa*	Hualong mountain, Ankang, Shaanxi, China	HL241	120424027	Y.Q. Zhang et al.
A. tonkinense Lecomte	*Lithocarpa*	Maolan, Guizhou, China	LB0954	LB0954	Y.F. Deng
A. sinopurpurascens W.C. Cheng	*Lithocarpa*	Zhongshan Bot. Garden, Nanjing, Jiangsu, China	NJ209	NJ209	Y. Xu et al.
A. sterculiaceum subsp. franchetii (Pax) A.E. Murray	*Lithocarpa*	Zhangjiajie, Hunan, China	QXJ107	WBGQXJ107	X.J. Qiao
A. davidii subsp. grosseri (Pax) P.C. de Jong	*Macrantha*	Baoji, Shaanxi, China	Ge131116	Ge131116	X.J. Ge
A. metcalfii Rehder	*Macrantha*	Wuzhi mountain, Ganzhou, Jiangxi, China	GN134	GN-134	X.J. Ge et al.
A. maximowiczii Pax	*Macrantha*	Hualong mountain, Ankang, Shaanxi, China	HL246	HS124	Y.Q. Zhang et al.
A. tegmentosum Maxim.	*Macrantha*	Changbai mountain, Jilin, China	JL049	CBS067	J. Ye

Table S5. (continued)

Species	Section	Locality	Sample ID	Voucher specimen	Collector
A. davidii subsp. grosseri (Pax) P.C. de Jong	*Macrantha*	Baoji, Shaanxi, China	Q032	Q032	S.L. Dong
A. maximowiczii Pax	*Macrantha*	Baoji, Shaanxi, China	Q151	Q151	S.L. Dong
A. davidii Franch.	*Macrantha*	Zhangjiajie, Hunan, China	QXJ070	WBGQXJ070	X.J. Qiao
A. davidii subsp. grosseri (Pax) P.C. de Jong	*Macrantha*	Xian Bot. Garden, Shaanxi, China	XA073	XA073	X.J. Ge
A. negundo L.	*Negundo*	Hangzhou, Zhejiang, China	HZ312	HZ312	Y. Xu et al.
A. henryi Pax	*Negundo*	Baoji, Shaanxi, China	Q163	Q163	S.L. Dong
A. henryi Pax	*Negundo*	Xian Bot. Garden, Shaanxi, China	XA019	XA019	X.J. Ge
A. coriaceifolium H. Lév.	*Oblonga*	Fuzhou, Fujian, China	FZ070	FZ070	Y. Xu et al.
A. oblongum Wall. ex DC.	*Oblonga*	Hanzhong, Shaanxi, China	Ge130922	Ge130922	X.J. Ge
A. coriaceifolium H. Lév.	*Oblonga*	Guilin Bot. Garden, Guangxi, China	GL120	GL120	Y. Xu et al.
A. sycopseoides Chun	*Oblonga*	Guilin Bot. Garden, Guangxi, China	GL333	GL333	Y. Xu et al.
A. oblongum Wall. ex DC.	*Oblonga*	Hualong mountain, Ankang, Shaanxi, China	HL238	ZP201705	Y.Q. Zhang et al.
A. lanceolatum Molliard	*Oblonga*	Maolan, Guizhou, China	LB0962	LB0962	Y.F. Deng
A. oblongum var. concolor Pax	*Oblonga*	Wuhan Bot. Garden, Hubei, China	X457	wh043	Y. Xu et al.
A. kwangsiense W.P. Fang & M.Y. Fang	*Palmata*	Guilin Bot. Garden, Guangxi, China	GL196	GL196	Y. Xu et al.
A. tutcheri Duthie	*Palmata*	Gannan Arboretum, Jiangxi, China	GN029	GN-029	X.J. Ge et al.
A. elegantulum W.P. Fang & P.L. Chiu	*Palmata*	Gannan Arboretum, Jiangxi, China	GN083	GN-083	X.J. Ge et al.
A. fabri Hance	*Palmata*	Gannan Arboretum, Jiangxi, China	GN092	GN-092	X.J. Ge et al.
A. tutcheri Duthie	*Palmata*	Gannan Arboretum, Jiangxi, China	GN100	GN-100	X.J. Ge et al.
A. olivaceum W.P. Fang & P.L. Chiu	*Palmata*	Gutian mountain, Quzhou, Zhejiang, China	GTS039	GTS QC 039	Z.X. Lai
A. japonicum Thunb.	*Palmata*	Hangzhou Bot. Garden, Zhejiang, China	HZ006	HZ006	Y. Xu et al.
A. pubinerve Rehder	*Palmata*	Hangzhou, Zhejiang, China	HZ328	HZ328	Y. Xu et al.
A. pseudosieboldianum (Pax) Kom.	*Palmata*	Changbai mountain, Jilin, China	JL020	CBS049	J. Ye

Table S5. (continued)

Species	Section	Locality	Sample ID	Voucher specimen	Collector
A. calcaratum Gagnep.	*Palmata*	Xishuangbanna, Yunnan, China	LQM0477	LQM0477	Q.M. Li
A. campbellii Hook.f. & Thomson ex Hiern	*Palmata*	Xishuangbanna, Yunnan, China	LQM1030	LQM1030	Y. Xu
A. fabri Hance	*Palmata*	Zhongshan Bot. Garden, Nanjing, Jiangsu, China	NJ056	NJ056	Y. Xu et al.
A. japonicum Thunb.	*Palmata*	Zhongshan Bot. Garden, Nanjing, Jiangsu, China	NJ175	NJ175	Y. Xu et al.
A. pauciflorum W.P. Fang	*Palmata*	Zhongshan Bot. Garden, Nanjing, Jiangsu, China	NJ207	NJ207	Y. Xu et al.
A. oliverianum Pax	*Palmata*	Zhongshan Bot. Garden, Nanjing, Jiangsu, China	NJ208	NJ208	Y. Xu et al.
A. linganense W.P. Fang & P.L. Chiu	*Palmata*	Zhongshan Bot. Garden, Nanjing, Jiangsu, China	NJ210	NJ210	Y. Xu et al.
A. sinense Pax	*Palmata*	Zhongshan Bot. Garden, Nanjing, Jiangsu, China	NJ217	NJ217	Y. Xu et al.
A. laevigatum Wall.	*Palmata*	Zhongshan Bot. Garden, Nanjing, Jiangsu, China	NJ245	NJ245	Y. Xu et al.
A. oliverianum Pax	*Palmata*	Baoji, Shaanxi, China	Q171	Q171	S.L. Dong
A. erianthum Schwer.	*Palmata*	Baoji, Shaanxi, China	Q269	Q269	S.L. Dong
A. duplicatoserratum var. chinense C. S. Chang	*Palmata*	Baoji, Shaanxi, China	Q350	Q350	S.L. Dong
A. flabellatum Rehder	*Palmata*	Hanzhong, Shaanxi, China	Q840	Q840	S.L. Dong
A. flabellatum Rehder	*Palmata*	Zhangjiajie, Hunan, China	QXJ162	WBGQXJ162	X.J. Qiao
A. ceriferum Rehder	*Palmata*	Zhangjiajie, Hunan, China	QXJ214	WBGQXJ214	X.J. Qiao
A. wilsonii Rehder	*Palmata*	Zhangjiajie, Hunan, China	QXJ215	WBGQXJ215	X.J. Qiao
A. wangchii W.P. Fang	*Palmata*	Hechi, Guangxi, China	S86	S86	T.J. Liu
A. duplicatoserratum var. chinense C. S. Chang	*Palmata*	Wuhan Bot. Garden, Hubei, China	X520	wh105	Y. Xu et al.
A. truncatum Bunge	*Platanoidea*	Pingshan, Hebei, China	Ge130727	Ge130727	X.J. Ge
A. shenkanense W.P. Fang ex C.C. Fu	*Platanoidea*	Baoji, Shaanxi, China	Ge131027	Ge131027	X.J. Ge
A. miaotaiense P.C. Tsoong	*Platanoidea*	Baoji, Shaanxi, China	Ge131047	Ge131047	X.J. Ge
A. shenkanense W.P. Fang ex C.C. Fu	*Platanoidea*	Baoji, Shaanxi, China	Ge131117	Ge131117	X.J. Ge
A. amplum subsp. catalpifolium (Rehder) Y.S. Chen	*Platanoidea*	Guilin Bot. Garden, Guangxi, China	GL203	GL203	Y. Xu et al.

Table S5. (continued)

**Species**	**Section**	**Locality**	**Sample ID**	**Voucher specimen**	**Collector**
A. campestre L.	*Platanoidea*	Hangzhou Bot. Garden, Zhejiang, China	HZ025	HZ025	Y. Xu et al.
A. truncatum Bunge	*Platanoidea*	Yichun, Heilongjiang, China	LSYL01	LSYL01	F. Jiang et al.
A. truncatum Bunge	*Platanoidea*	Zhongshan Bot. Garden, Nanjing, Jiangsu, China	NJ133	NJ133	Y. Xu et al.
A. truncatum Bunge	*Platanoidea*	Zhongshan Bot. Garden, Nanjing, Jiangsu, China	NJ212	NJ212	Y. Xu et al.
A. mayrii Schwer.	*Platanoidea*	Zhongshan Bot. Garden, Nanjing, Jiangsu, China	NJ213	NJ213	Y. Xu et al.
A. miaotaiense P.C. Tsoong	*Platanoidea*	Baoji, Shaanxi, China	Q128	Q128	S.L. Dong
A. pictum subsp. mono (Maxim.) H. Ohashi	*Platanoidea*	Baoji, Shaanxi, China	Q150	Q150	S.L. Dong
A. amplum Rehder	*Platanoidea*	Zhangjiajie, Hunan, China	QXJ020	WBGQXJ020	X.J. Qiao
A. amplum Rehder	*Platanoidea*	Zhangjiajie, Hunan, China	QXJ103	WBGQXJ103	X.J. Qiao
A. miaotaiense P.C. Tsoong	*Platanoidea*	Xian Bot. Garden, Shaanxi, China	XA023	XA023	X.J. Ge
A. ukurunduense Trautv. & C.A. Mey.	*Spicata*	Yichun, Heilongjiang, China	LSYL28	LSYL28	F. Jiang et al.
A. caudatum Wall.	*Spicata*	Baoji, Shaanxi, China	Q456	Q456	S.L. Dong
A. caudatum Wall.	*Spicata*	Baoji, Shaanxi, China	Q700	Q700	S.L. Dong
A. mandshuricum Maxim.	*Trifoliata*	Changbai mountain, Jilin, China	CBS004	CBS004	J. Ye
A. nikoense (Miq.) Maxim.	*Trifoliata*	Hangzhou Bot. Garden, Zhejiang, China	HZ200	HZ200	Y. Xu et al.
A. triflorum Kom.	*Trifoliata*	Changbai mountain, Jilin, China	JL029	CBS064	J. Ye
A. griseum (Franch.) Pax	*Trifoliata*	Ankang, Shaanxi, China	Q888	Q888	S.L. Dong
A. griseum (Franch.) Pax	*Trifoliata*	Wuhan Bot. Garden, Hubei, China	X422	wh008	Y. Xu et al.

Table S6. The comparison between nrDNA datasets that with and without ETS.

Data set	Aligned length (bp)	Length range (bp)	Variable sites		PI sites		SDE in tree-based method
			Number	%	Number	%	
partial ETS	834	308-778	113	13.55	94	11.27	NA
nrDNA without ETS	5,939	5,810-5,856	255	4.29	203	3.42	71.42% (15/21)
nrDNA	6,773	6,154-6,625	368	5.43	297	4.39	80.95% (17/21)

ETS: external transcribed spacer; SDE：species discriminatory efficiency. NA: not assessed.

Table S7. Plastomes downloaded from Genbank and their accession numbers.

No.	Species	Accession	No.	Species	Accession
1	*Acer acuminatum*	MN864496	94	*A. oliverianum*	KY827815
2	*A. amplum*	MH784399	95	*A. oliverianum*	MW067060
3	*A. amplum*	MK648114	96	*A. oliverianum*	NC_051958
4	*A. amplum*	MW067026	97	*A. opalus*	NC_056221
5	*A. amplum*	MW493345	98	*A. opalus* subsp. *obtusatum*	MW067057
6	*A. amplum*	NC_050668	99	*A. paihengii*	MZ934750
7	*A. amplum* subsp. *catalpifolium*	MK479218	100	*A. palmatum*	MK578668
8	*A. amplum* subsp. *catalpifolium*	MW067027	101	*A. palmatum*	MW599990
9	*A. amplum* subsp. *catalpifolium*	NC_041080	102	*A. palmatum*	NC_034932
10	*A. argutum*	NC_056202	103	*A. palmatum* var. *palmatum*	MN864504
11	*A. buergerianum*	MW067029	104	*A. pauciflorum*	NC_056222
12	*A. buergerianum*	NC_034744	105	*A. paxii*	MK479215
13	*A. buergerianum* subsp. *ningpoense*	KF753631	106	*A. paxii*	NC_056223
14	*A. caesium*	MW067030	107	*A. pectinatum*	MW067064
15	*A. caesium*	NC_051955	108	*A. pectinatum*	NC_050672
16	*A. caesium* subsp. *giraldii*	MK479225	109	*A. pensylvanicum*	NC_056224
17	*A. calcaratum*	NC_056203	110	*A. pentaphyllum*	MK341079
18	*A. campbellii*	MW801166	111	*A. pentaphyllum*	MK479216
19	*A. campestre*	NC_056204	112	*A. pentaphyllum*	MN315278
20	*A. cappadocicum*	MK479217	113	*A. pentaphyllum*	MN661390
21	*A. cappadocicum*	NC_051956	114	*A. pentaphyllum*	MN864505
22	*A. cappadocicum* subsp. *divergens*	MW067034	115	*A. pentaphyllum*	NC_049167
23	*A. cappadocicum* subsp. *sinicum*	MW067035	116	*A. pictum* subsp. *mono*	MK479227
24	*A. carpinifolium*	MN864497	117	*A. pictum* subsp. *mono*	NC_049127
25	*A. caudatifolium*	MK479219	118	*A. pilosum*	MN864506
26	*A. caudatifolium*	MK479226	119	*A. platanoides*	NC_051959
27	*A. ceriferum*	MW800986	120	*A. pseudoplatanus*	MN864508
28	*A. cinnamomifolium*	MK479220	121	*A. pseudosieboldianum*	MW067066
29	*A. cinnamomifolium*	MT752969	122	*A. pseudosieboldianum*	NC_046487
30	*A. cinnamomifolium*	NC_056164	123	*A. pseudosieboldianum* var. *microsieboldianum*	MW067067
31	*A. circinatum*	NC_056205	124	*A. pubipetiolatum* var. *pingpienense*	MZ944848
32	*A. cissifolium*	MW067037	125	*A. pycnanthum*	NC_056225
33	*A. cissifolium*	NC_049162	126	*A. robustum*	MK479212
34	*A. cordatum*	MW800944	127	*A. rubescens*	NC_056226
35	*A. coriaceifolium*	NC_050669	128	*A. rubrum*	MN864509
36	*A. crataegifolium*	NC_056206	129	*A. rufinerve*	NC_056227
37	*A. davidii*	MK193784	130	*A. saccharinum*	NC_056228
38	*A. davidii*	MK193785	131	*A. saccharum*	NC_051960
39	*A. davidii*	MK193786	132	*A. saccharum* subsp. *floridanum*	MW067072
40	*A. davidii*	MW800922	133	*A. saccharum* subsp. *leucoderme*	MW067073
41	*A. davidii*	NC_030331	134	*A. saccharum* subsp. *nigrum*	MW067074
42	*A. diabolicum*	NC_056207	135	*A. saccharum* subsp. *saccharum*	MW067075
43	*A. distylum*	NC_056209	136	*A. saccharum* subsp. *skutchii*	MW067080
44	*A. elegantulum*	MW801101	137	*A. sempervirens*	NC_056229
45	*A. elegantulum*	NC_056210	138	*A. serrulatum*	NC_056230
46	*A. fabri*	MW067044	139	*A. shenkanense*	NC_056231
47	*A. fabri*	MZ664553	140	*A. sikkimense*	NC_056232
48	*A. fabri*	NC_050670	141	*A. sinense*	NC_057529
49	*A. fenzelianum*	MN315273	142	*A. sino-oblongum*	NC_040106
50	*A. fenzelianum*	NC_045527	143	*A. stachyophyllum*	NC_051961

Table S7. (continued)

No.	Species	Accession	No.	Species	Accession
51	*A. flabellatum*	MF787384	144	*A. stachyophyllum* subsp. *betulifolium*	MW067082
52	*A. flabellatum*	MK479222	145	*A. stachyophyllum* subsp. *stachyophyllum*	MW067081
53	*A. flabellatum*	MW067032	146	*A. sterculiaceum*	NC_050673
54	*A. flabellatum*	NC_050671	147	*A. sterculiaceum* subsp. *franchetii*	MN315284
55	*A. glabrum*	MN864498	148	*A. sterculiaceum* subsp. *franchetii*	MW067083
56	*A. granatense*	NC_056211	149	*A. sterculiaceum* subsp. *franchetii*	MW067093
57	*A. grandidentatum*	NC_056212	150	*A. sterculiaceum* subsp. *franchetii*	MW800946
58	*A. griseum*	MT355071	151	*A. sterculiaceum* subsp. *sterculiaceum*	MN864510
59	*A. griseum*	MT355072	152	*A. sutchuenense* subsp. *tienchuanense*	NC_049166
60	*A. griseum*	MT355073	153	*A. takesimense*	MW067084
61	*A. griseum*	MW067047	154	*A. takesimense*	NC_046488
62	*A. griseum*	NC_034346	155	*A. tataricum*	NC_050674
63	*A. henryi*	MW067048	156	*A. tataricum* subsp. *aidzuense*	MW067085
64	*A. henryi*	NC_049163	157	*A. tataricum* subsp. *ginnala*	MK479223
65	*A. hyrcanum*	NC_056213	158	*A. tataricum* subsp. *ginnala*	MK479224
66	*A. japonicum*	NC_056214	159	*A. tataricum* subsp. *ginnala*	MN790641
67	*A. laevigatum*	NC_042443	160	*A. tataricum* subsp. *ginnala*	MN864511
68	*A. laurinum*	NC_056215	161	*A. tataricum* subsp. *semenovii*	MW067086
69	*A. leipoense*	MZ043780	162	*A. tataricum* subsp. *tataricum*	MW067087
70	*A. leipoense*	MZ725939	163	*A. tegmentosum*	MK942342
71	*A. lobelii*	NC_056216	164	*A. tegmentosum*	NC_056233
72	*A. longipes*	MW067053	165	*A. tenuifolium*	NC_056234
73	*A. longipes*	NC_049126	166	*A. tetramerum*	MK479228
74	*A. lucidum*	MK479214	167	*A. trautvetteri*	NC_056235
75	*A. macrophyllum*	NC_056217	168	*A. triflorum*	MW067091
76	*A. mandshuricum*	MW067055	169	*A. triflorum*	NC_047296
77	*A. mandshuricum*	NC_049164	170	*A. truncatum*	MH638284
78	*A. miaotaiense*	NC_030343	171	*A. truncatum*	MH716034
79	*A. micranthum*	MN864500	172	*A. truncatum*	MK479213
80	*A. monspessulanum*	NC_056218	173	*A. truncatum*	NC_037211
81	*A. morrisonense*	NC_029371	174	*A. tsinglingense*	MN393475
82	*A. negundo*	MN841452	175	*A. tutcheri*	MW067094
83	*A. negundo*	MN864501	176	*A. tutcheri*	NC_051542
84	*A. negundo*	MT336153	177	*A. ukurunduense*	NC_060654
85	*A. negundo*	NC_051957	178	*A. velutinum*	NC_056236
86	*A. nikoense*	MN864499	179	*A. wilsonii*	MW067096
87	*A. nikoense*	NC_049165	180	*A. wilsonii*	NC_040988
88	*A. nipponicum*	MN864502	181	*A. yangbiense*	CM017775
89	*A. oblongum*	MN864503	182	*A. yangbiense*	MK479229
90	*A. oblongum*	MZ073338	183	*A. yangbiense*	NC_050351
91	*A. oblongum*	NC_056208	184	*A. yangjuechi*	NC_053258
92	*A. obtusifolium*	NC_056219	185	*Dimocarpus longan*	MW067098
93	*A. olivaceum*	NC_056220	186	*Litchi chinensis*	MW067100

Table S8. Genbank accession numbers of *Acer* plant materials generated in this study.

**Species**	**Sample ID**	**SRA accession**	**Plastome accession**	**nrDNA accession**
Acer amplum	QXJ020	SRR21071082	OP219592	OP221123
A. amplum	QXJ103	SRR21071079	OP219594	OP221125
A. amplum subsp. *catalpifolium*	GL203	SRR21071145	OP219534	OP221065
A. barbinerve	JL008	SRR21071128	OP219550	OP221081
A. calcaratum	LQM0477	SRR21071118	OP219559	OP221090
A. campbellii	LQM1030	SRR21071117	OP219560	OP221091
A. campestre	HZ025	SRR21071132	OP219546	OP221077
A. carpinifolium	NJ216	SRR21071102	OP219573	OP221104
A. caudatum	Q456	SRR21071086	OP219588	OP221119
A. caudatum	Q700	SRR21071085	OP219589	OP221120
A. ceriferum	QXJ214	SRR21071076	OP219597	OP221128
A. coriaceifolium	FZ070	SRR21071147	OP219524	OP221055
A. coriaceifolium	GL120	SRR21071066	OP219532	OP221063
A. davidii	QXJ070	SRR21071080	OP219593	OP221124
A. davidii subsp. *grosseri*	Ge131116	SRR21071092	OP219530	OP221061
A. davidii subsp. *grosseri*	Q032	SRR21071099	OP219576	OP221107
A. davidii subsp. *grosseri*	XA073	SRR21071067	OP219605	OP221136
A. duplicatoserratum var. *chinense*	Q350	SRR21071087	OP219587	OP221118
A. duplicatoserratum var. *chinense*	X520	SRR21071071	OP219602	OP221133
A. elegantulum	GN083	SRR21071142	OP219537	OP221068
A. erianthum	Q269	SRR21071088	OP219586	OP221117
A. fabri	GN092	SRR21071141	OP219538	OP221069
A. fabri	NJ056	SRR21071113	OP219563	OP221094
A. flabellatum	Q840	SRR21071084	OP219590	OP221121
A. flabellatum	QXJ162	SRR21071077	OP219596	OP221127
A. griseum	Q888	SRR21071083	OP219591	OP221122
A. griseum	X422	SRR21071073	OP219600	OP221131
A. henryi	Q163	SRR21071093	OP219582	OP221113
A. henryi	XA019	SRR21071069	OP219603	OP221134
A. japonicum	HZ006	SRR21071133	OP219545	OP221076
A. japonicum	NJ175	SRR21071110	OP219566	OP221097
A. kwangsiense	GL196	SRR21071146	OP219533	OP221064
A. laevigatum	NJ245	SRR21071100	OP219575	OP221106
A. lanceolatum	LB0962	SRR21071121	OP219556	OP221087
A. laurinum	LQM0060	SRR21071120	OP219557	OP221088
A. laurinum	LQM0426	SRR21071119	OP219558	OP221089
A. linganense	NJ210	SRR21071106	OP219570	OP221101
A. mandshuricum	CBS004	SRR21071148	OP219523	OP221054
A. maximowiczii	HL246	SRR21071134	OP219544	OP221075
A. maximowiczii	Q151	SRR21071095	OP219580	OP221111
A. mayrii	NJ213	SRR21071104	OP219572	OP221103
A. metcalfii	GN134	SRR21071139	OP219540	OP221071

Table S8. (continued)

Species	Sample ID	SRA accession	Plastome accession	nrDNA accession
A. miaotaiense	Ge131047	SRR21071103	OP219529	OP221060
A. miaotaiense	Q128	SRR21071097	OP219578	OP221109
A. miaotaiense	XA023	SRR21071068	OP219604	OP221135
A. negundo	HZ312	SRR21071130	OP219548	OP221079
A. nikoense	HZ200	SRR21071131	OP219547	OP221078
A. oblongum	Ge130922	SRR21071125	OP219527	OP221058
A. oblongum	HL238	SRR21071137	OP219542	OP221073
A. oblongum var. *concolor*	X457	SRR21071072	OP219601	OP221132
A. olivaceum	GTS039	SRR21071138	OP219541	OP221072
A. oliverianum	NJ208	SRR21071108	OP219568	OP221099
A. oliverianum	Q171	SRR21071091	OP219583	OP221114
A. pauciflorum	NJ207	SRR21071109	OP219567	OP221098
A. pictum subsp. *mono*	Q150	SRR21071096	OP219579	OP221110
A. pseudoplatanus	Ge19021	SRR21071070	OP219525	OP221056
A. pseudosieboldianum	JL020	SRR21071127	OP219551	OP221082
A. pubinerve	HZ328	SRR21071129	OP219549	OP221080
A. shenkanense	Ge131027	SRR21071114	OP219528	OP221059
A. shenkanense	Ge131117	SRR21071081	OP219531	OP221062
A. sinense	NJ217	SRR21071101	OP219574	OP221105
A. sinopurpurascens	NJ209	SRR21071107	OP219569	OP221100
A. stachyophyllum subsp. *betulifolium*	Q152	SRR21071094	OP219581	OP221112
A. stachyophyllum subsp. *betulifolium*	Q264	SRR21071090	OP219584	OP221115
A. stachyophyllum subsp. *betulifolium*	Q265	SRR21071089	OP219585	OP221116
A. sterculiaceum subsp. *franchetii*	HL241	SRR21071135	OP219543	OP221074
A. sterculiaceum subsp. *franchetii*	QXJ107	SRR21071078	OP219595	OP221126
A. sycopseoides	GL333	SRR21071144	OP219535	OP221066
A. tataricum subsp. *ginnala*	JL054	SRR21071123	OP219554	OP221085
A. tataricum subsp. *theiferum*	NJ128	SRR21071112	OP219564	OP221095
A. tataricum subsp. *theiferum*	Q105	SRR21071098	OP219577	OP221108
A. tegmentosum	JL049	SRR21071124	OP219553	OP221084
A. tonkinense	LB0954	SRR21071122	OP219555	OP221086
A. triflorum	JL029	SRR21071126	OP219552	OP221083
A. truncatum	Ge130727	SRR21071136	OP219526	OP221057
A. truncatum	LSYL01	SRR21071116	OP219561	OP221092
A. truncatum	NJ133	SRR21071111	OP219565	OP221096
A. truncatum	NJ212	SRR21071105	OP219571	OP221102
A. tutcheri	GN029	SRR21071143	OP219536	OP221067
A. tutcheri	GN100	SRR21071140	OP219539	OP221070
A. ukurunduense	LSYL28	SRR21071115	OP219562	OP221093
A. wangchii	S86	SRR21071074	OP219599	OP221130
A. wilsonii	QXJ215	SRR21071075	OP219598	OP221129

Table S9. The partition scheme of 80 coding sequences (CDSs).

Subset	Best Model	Partition scheme
1	GTR+I+G	*rps16, accD, atpF, rpl33*
2	GTR+I+G	*rpoC1, atpA, rpl16, ndhH, rpoB*
3	GTR+I+G	*clpP, ndhJ, rps11, atpB, psbN*
4	GTR+G	*petA, rpl14, atpE, rpl20, rps14*
5	GTR+I+G	*psbB, psbC, atpH, psbH*
6	TVM+I+G	*ndhE, atpI, psbZ, ndhK, petD, psaJ, psbI*
7	TVM+I+G	*ccsA, ycf4, ndhD*
8	TVM+I+G	*cemA, ndhA*
9	TVM+I+G	*matK*
10	TVM	*ndhB, psbF*
11	GTR+G	*petL, psbT, ndhG, ndhC, psbK*
12	TVM+I+G	*ndhF*
13	GTR+I+G	*ndhI, psbM*
14	HKY	*orf42*
15	GTR+I+G	*psaC, psbE, psbJ, psbD, petB, psaA, psaB*
16	HKY	*petN, petG*
17	TRN+I+G	*psaI, psbL*
18	GTR+I+G	*psbA*
19	GTR+I+G	*rbcL*
20	TVM+G	*rpl22*
21	K81UF	*rps12, rpl2, rps7, rpl23*
22	TIM+I+G	*rpl32*
23	TVM+I	*rpl36, ycf3*
24	GTR+I+G	*rpoA, rps3, rps15*
25	TVM+I+G	*rpoC2*
26	TVM+I	*rps4, rps18*
27	TVM+I+G	*rps8, rps19*
28	TVM+I+G	*rps2*
29	K81	*ycf15*
30	TVM+I+G	*ycf1*
31	TVM+I+G	*ycf2*
